# Supplementary material for: Somatic SF3B1 hotspot mutation in prolactinomas
Source: Nat Commun. 2020 May 19;11:2506. doi: 10.1038/s41467-020-16052-8 (PMC7237453; doi:10.1038/s41467-020-16052-8)
Supplement: Supplementary file 1 — Supplementary Information [file 41467_2020_16052_MOESM1_ESM.pdf]

Supplementary Information for

Somatic *SF3B1* Hotspot Mutation in Prolactinomas

Authors: Chuzhong Li[1, 2, 3, 4]†, Weiyan Xie[1]†, Jared Rosenblum[5], Jianyu Zhou[6], Jing Guo[1, 2], Yazhou Miao[1, 2], Yutao Shen[1, 2], Hongyun Wang[1], Lei Gong[1], Mingxuan Li[1, 2], Sida Zhao[1, 2], Sen Cheng[1, 2], Haibo Zhu[1, 2], Tao Jiang[6, 7], Shiying Ling[8], Fei Wang[8], Hongwei Zhang[9], Mingshan Zhang[9], Yanming Qu[9], Qi Zhang[5], Guilin Li[10], Junmei Wang[10], Jun Ma[11], Zhengping Zhuang[5, 12*] & Yazhuo Zhang[1, 2, 3, 4*]

* Correspondence to: zhangyazhuo@ccmu.edu.cn; zhengping.zhuang@nih.gov

†These authors contributed equally to this work

Materials and Methods

High Throughput Sequencing

Whole genome sequencing of DNA extracted from 21 frozen tumors and matched peripheral blood was performed to investigate somatic alterations. Tiers for tumor-specific mutations were designated and annotated based on the methodological rigor for mutation calling. In order to determine gene expression level and alterative splicing events, transcriptome sequencing was conducted for 15 tumors and 10 normal tissues.

DNA and RNA extractions

The genomic DNA were extracted from frozen tissue species and peripheral blood using the AllPrep DNA/RNA Mini kit (80204, Qiagen) and DNeasy blood & tissue kit (69504, Qiagen), respectively. The genomic DNA were extracted from formalin-fixed, paraffin-embedded tumor tissues species and peripheral blood using the GeneRead DNA FFPE Kit (180134, Qiagen). The GeneRead DNA FFPE Kit provides a streamlined procedure that includes the removal of deaminated cytosine to prevent false results in DNA sequencing.

Total amount of 500ng DNA with high-molecular weight (>20Kb single band) was used for the library preparation. For RNA extractions, tissue sections from same patient and normal tissues from unrelated person were performed with the AllPrep DNA/RNA Mini kit according to the instructions of the manufacture. RNA degradation and contamination were monitored on 1% agarose gels. The RNA quality and quantity were assessed at the RNA Nano 6000 Assay Kit of the Bioanalyzer 2100 system (Aligent Technologies, USA). Samples with RNA integrity number (RIN) of over 6.8 were further analyzed by RNA-seq.

Whole genome sequencing and variant detection

DNA and RNA sequencing and integrative analysis of omics data in this study were completed by Novogene Bioinformatics Institute. Sequencing library was constructed using Truseq Nano DNA HT Sample Prep Kit (FC-121-4003, Illumina) and sequenced on Illumina HiSeq X platform to an average depth of 54x for tumors and 34x for matched normals, with 99.49% of the known genome being covered. After the exclusion of reads containing adapter

contamination and low-quality/unrecognizable nucleotides, clean data was mapped to the reference human genome (UCSC hg19) by Burrows-Wheeler Aligner (BWA) software[1] to get the original mapping results stored in BAM format. SAMtools[2], Picard (http://broadinstitute.github.io/picard/), and GATK[3] were used to sort BAM files and do base quality recalibration, duplicate removing and local realignment to generate final BAM file for mutation calling. Somatic single nucleotide variations (SNVs) and small insertions and deletions (InDels) from paired tumor-normal samples were identified by MuTect [4] and Strelka[5] with default parameters.

Four post-processing filters were applied to the set of somatic variants detected by both algorithms to improve specificity as follows: (1) The mutation position should be covered by at least 5 reads in both tumors and their normal pairs; (2) Mutant alleles coverage in tumors should be more than 4X. Specifically, if the reads supporting variant alleles in tumors are more than 6, the allelic fraction should be more than 10%, otherwise, the allelic fraction in excess of 15% is necessary; (3) Alternate alleles observed in single directions of reads were excluded to ameliorate PCR artifact bias; (4) Putative somatic variants were cross-referenced with the panel of normal samples to identify calls coinciding with high error rate loci. The candidate present in two or more germline normal samples with at least 3 mutation-carrying reads was removed. Somatic variants were manually interrogated for accuracy by Integrative Genomics Viewer. We binned SNVs into three tiers. Tier1 contains SNVs validated by Sanger sequencing, Time of Flight Mass Spectrometer (TOF) or RNA-seq. Tier2 contains the remaining SNVs passing the four post-processing filters described before and meet tier1 criteria. Tier3 contains all somatic nonsynonymous substitutions in the amino acid coding regions identified by MuTect and Strelka, and those meet tier2 criteria. The landscape is based on Tier2 for accuracy and comprehensiveness of mutation calling.

RNA-Seq data processing and analysis

Raw data of fastq format were firstly processed in-house perl scripts and clean data were obtained by removing reads containing adapters, reads containing ploy-N and low-quality

reads. All the downstream analyses were based on the clean data with high quality. Paired-end RNA-seq clean reads were mapped to the human reference genome (NCBI37/hg19) using TopHat2[6]. The mapped reads of each sample were assembled by Cufflinks (v2.1.1)[7] in a reference-based approach. Cuffdiff (v2.1.1) (http://cole-trapnell-lab.github.io/cufflinks/cuffdiff/index.html) was used to determine the expression levels of both lncRNAs and coding genes in each sample. The expression is represented as FPKM values. FPKM means fragments per kilo-base of exon per million fragments mapped, calculated based on the length of the fragments and reads count mapped to this fragment.

Splicing reporter assays

A 515 kb region of the human ESRRG gene contains a fragment of the ESRRG gene spanning exons 5 and includes 353 bp of intron 4 sequences in this region, was amplified using the following primers: 5’-gtgccaaaatgtaaataacattc-3’, 5’-ctttgtattaatctacatgga-3’, 5’-aaagtagaagtatttgctagga-3’, 5’-aactatctgaaaattcaactc-3’， 5’-ggtaggtaccctacttcaccaaatcccacttaag-3’, 5’-tagtggatccgcctggaatatgcttcgcccatcc-3’. The fragment was cloned into pcMINI-C. The splicing reporters were transfected into MCF7 cells and RNA were harvested after 36 h. Spliced products were amplified using the following primers: 5’-ctagagaacccactgcttac-3’, 5’-tagaaggcacagtcgagg-3’. This strategy amplified minigene-derived transcripts but not endogenous ESRRG mRNA. PCR products were analyzed on either 2% agarose gels. All PCR products were confirmed by DNA sequencing.

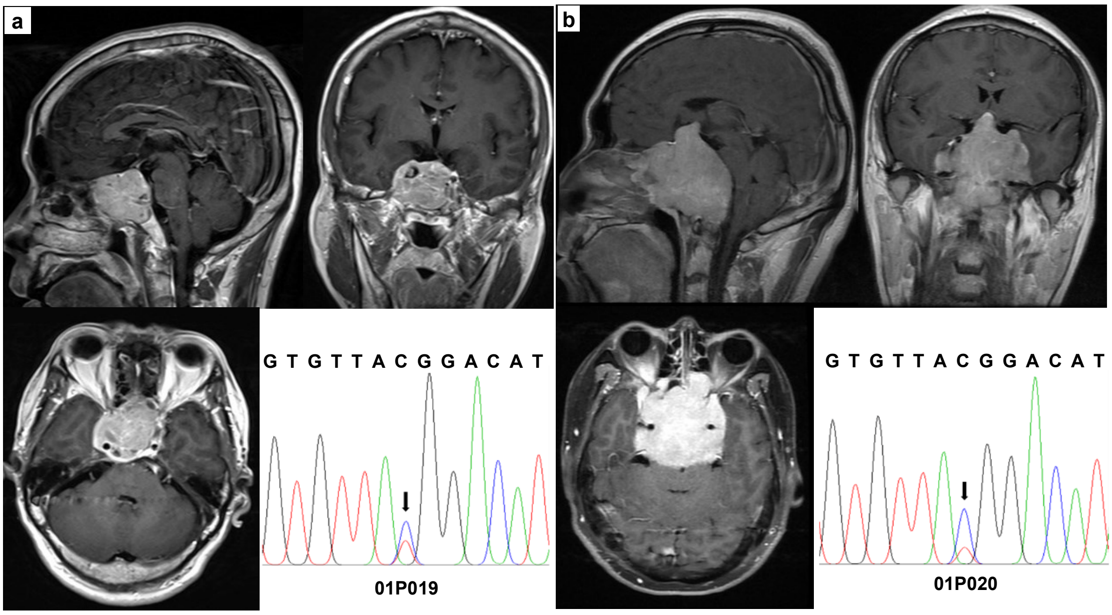

Supplementary Figure 1 Two patients with *SF3B1* mutation detected from whole genome sequencing.

a, b Brain MRI of two patients with *SF3B1* mutation detected from whole genome sequencing and validation by Sanger sequencing. Arrows indicate mutated bases.

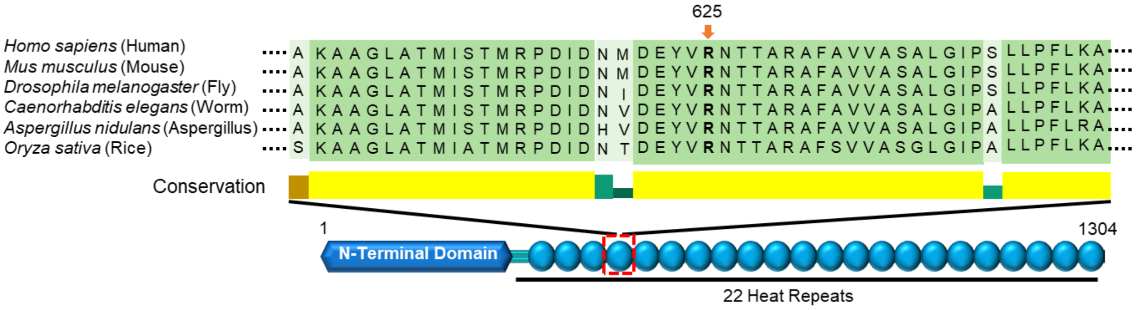

Supplementary Figure 2 Schematic representation of the SF3B1 protein with the primary structural domains highlighted.

R625H hotspot mutations affect the HEAT domains (Huntingtin, elongation factor 3, protein phosphatase 2A, TOR1). Protein sequence alignments of the SF3B1 C-terminal domain are around the altered residues (R625H), conserved in evolutionarily diverse species.

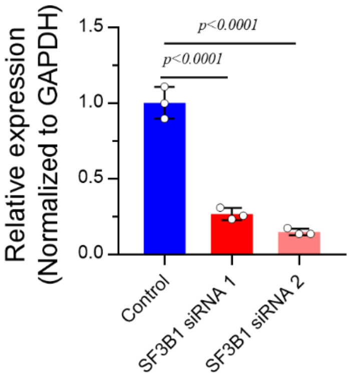

Supplementary Figure 3 Representative qRT-PCR for SF3B1 expression levels in primary human prolactinoma cells transfected with control or specific SF3B1 siRNA. GAPDH was used as internal control. Results are displayed as mean ± SD (n=3 per group). The p-values by one-way ANOVA followed by Dunnett's multiple comparisons test are indicated. Source data are provided as a Source Data file.

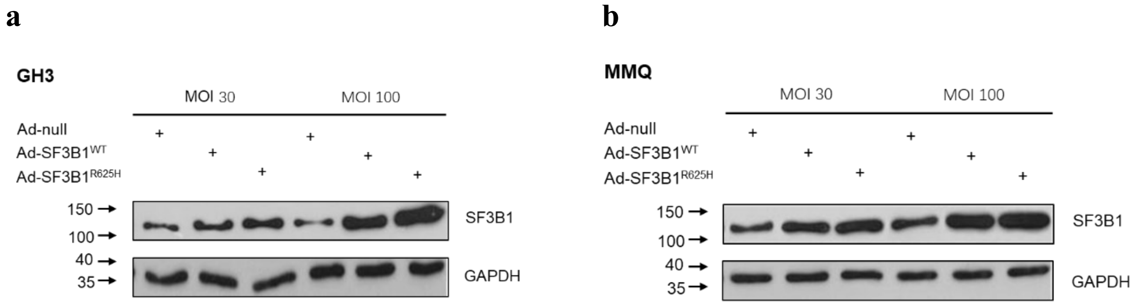

Supplementary Figure 4 Western blot analysis of SF3B1.

a, Representative western blot analysis of SF3B1 in GH3 infected with Ad-null (used as the control) or Ad-SF3B1^{WT} or Ad-SF3B1^{R625H} at the indicated MOI. **b**, Representative western blot analysis of SF3B1 in MMQ infected with Ad-null (used as the control) or Ad-SF3B1^{WT} or Ad-SF3B1^{R625H} at the indicated MOI.

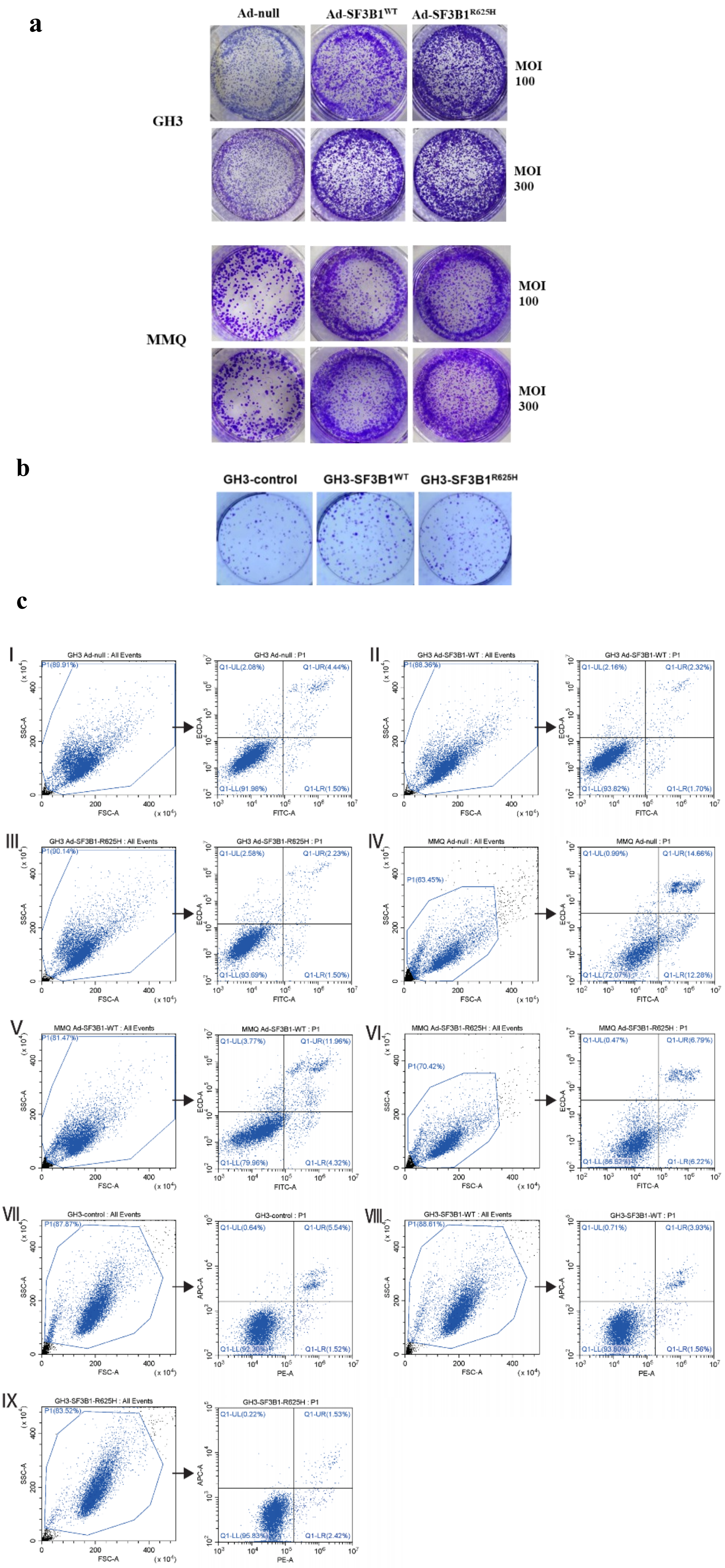
a
Ad-null
Ad-SF3B1^{WT}
Ad-SF3B1^{R625H}
GH3
MMQ
MOI 100
MOI 300
b
GH3-control
GH3-SF3B1^{WT}
GH3-SF3B1^{R625H}
c
I
II
III
IV
V
VI
VII
VIII
IX

Supplementary Figure 5 SF3B1^{R625H} enhances proliferation and suppress apoptosis of GH3 and MMQ cells.

a, The focus formation of GH3/MMQ cells transduced with Ad-null, Ad-SF3B1^{WT} and Ad-SF3B1^{R625H}. **b**, The focus formation of stable GH3-control, GH3- SF3B1^{WT}, GH3-SF3B1^{R625H} cells. c, Annexin V/PI staining and flow cytometry showed the percentages of apoptosis of GH3/MMQ cells transduced with control, SF3B1^{WT} and SF3B1^{R625H}. Ⅰ-Ⅲ, Ad-GH3 cells. Ⅳ-Ⅵ, Ad-MMQ cells. Ⅶ-Ⅸ, stable GH3 cells.

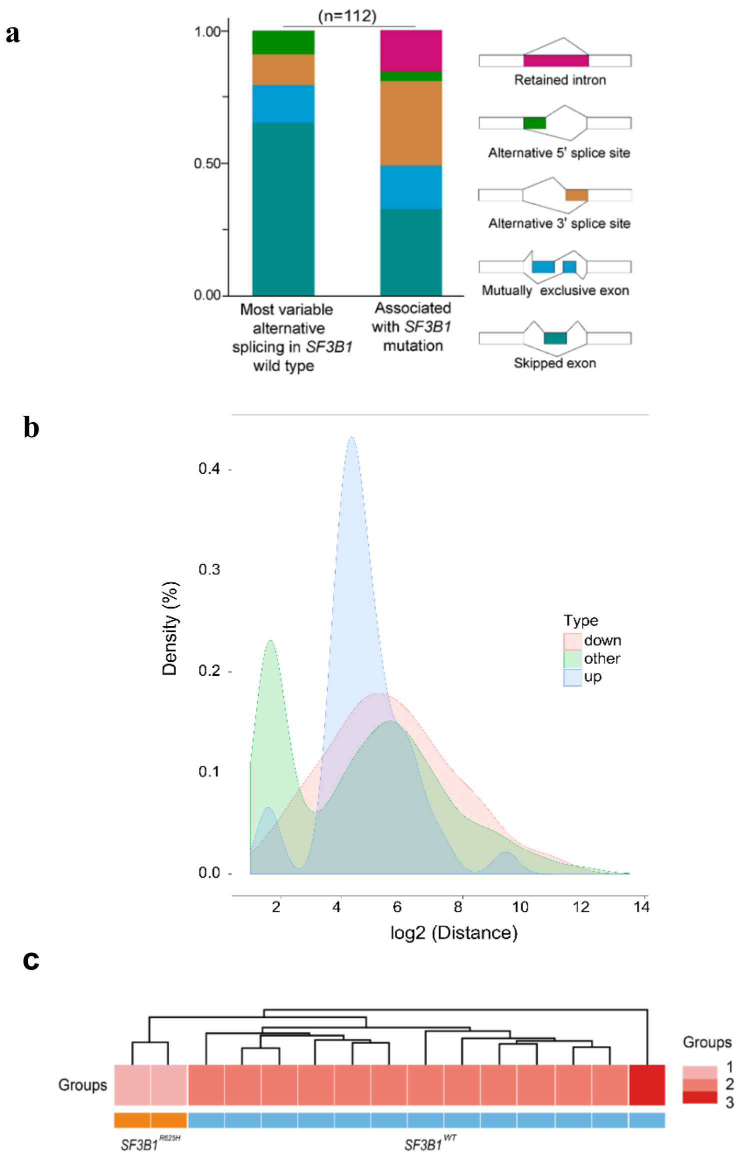

Supplementary Figure 6 Alternative splicing in *SF3B1* mutant prolactinomas.

a, Categories of 112 alternative splicing events significantly associated with *SF3B1*^{R625H} mutations in prolactinomas (right) compared with the 112 most variable splicing events in *SF3B1* wild type samples (left) from RNA-seq datasets. **b**, Density plot of the relative positions of cryptic 3'ss compared to their canonical splice sites (presented as log2 of distance between competing 3’ss) in *SF3B1* mutant tissues. Blue line: upstream cryptic sites. Red line: downstream cryptic sites. Green line: A3SS unaffected by *SF3B1* mutations. **c**, Unsupervised hierarchical clustering of 15 prolactinomas. Different colors of groups represent clustering subgroups based on the most variable alternative splicing events.

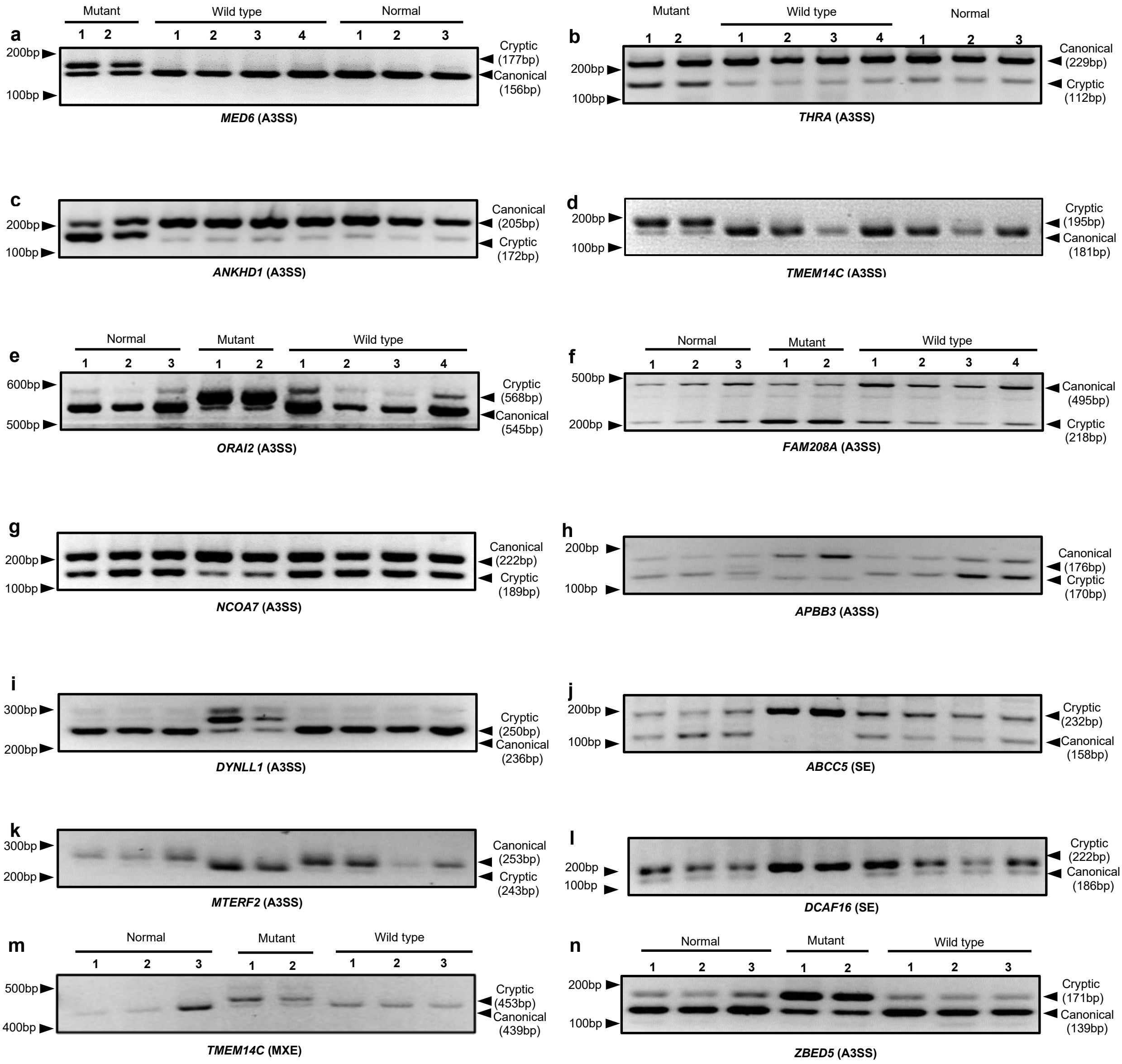

Supplementary Figure 7 Alternative splicing in *SF3B1* mutant prolactinomas.

a-n, PCR products of the 13 genes (not including *ESRRG*) – *MED6,* *THRA*, *ANKHD1*, *TMEM14C (A3SS)*, *ORAI2*, *FAM208A*, *NCOA7*, *APBB3*, *DYNLL1*, *ABCC5*, *MTERF2*, *DCAF16*, *TMEM14C (MXE)* and *ZBED5* were amplified from *SF3B1*-mutant prolactinoma samples (Mutant), Wild type prolactinoma samples and healthy control samples (Normal). The cryptic and canonical transcripts were indicated by the arrow.

a

Gene	Motifs
ESRRG	3088
ANKHD1	943
NCOA7	605
ABCC5	414
FAM208A	414
ZBED5	207
MED6	117
DYNLL1	100
THRA	71
DCAF16	45
MTERF2	40
TMEM14C	14
APBB3	12
ORAI2	7

b

Transcript	Intron	Intron Ending with U2AF2 Motif	Percentage
**NM_001134285.2**	8	5	62.5%
**NM_001243505.1**	6	3	50.0%
**NM_001243506.1**	6	3	50.0%
**NM_001243507.1**	7	4	57.1%
**NM_001243509.1**	8	5	62.5%
**NM_001243510.2**	8	5	62.5%
**NM_001243511.2**	7	4	57.1%
**NM_001243512.1**	8	5	62.5%
**NM_001243513.1**	7	4	57.1%
**NM_001243514.1**	6	3	50.0%
**NM_001243515.1**	8	5	62.5%
**NM_001243518.1**	7	4	57.1%
**NM_001243519.1**	8	5	62.5%
**NM_001438.3**	6	3	50.0%
**NM_206594.2**	8	5	62.5%
**NM_206595.2**	7	4	57.1%

c

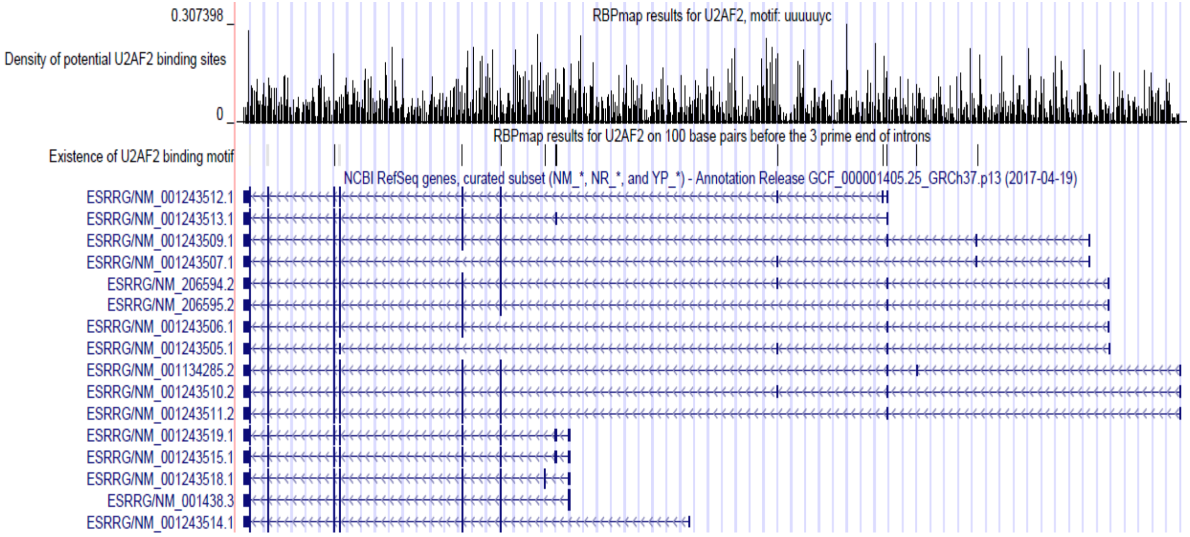

d

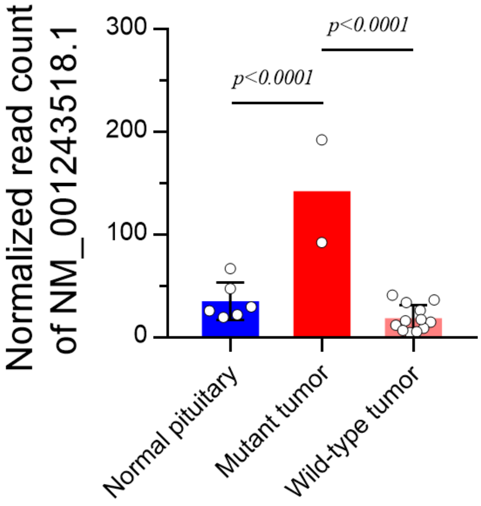

e

Transcript	Wild type *vs* Normal Base Mean	Wild type *vs* Normal Log_2 FC	Wild type *vs* Normal P Value	Wild type *vs* Normal P Adj	Mutant *vs* Normal Base Mean	Mutant *vs* Normal Log_2 FC	Mutant *vs* Normal P Value	Mutant *vs* Normal P Adj
NM_001134285.2	169.399	-0.444	0.027	0.128	230.203	-0.010	0.966	0.990
NM_001243505.1	8.136	-0.813	0.296	0.562	15.964	0.984	0.682	0.897
NM_001243506.1	0.294	1.506	0.640	-	0.000	-	-	-
NM_001243507.1	14.470	3.519	0.000	0.002	8.892	3.880	0.232	0.541
NM_001243509.1	42.568	-0.046	0.937	0.976	54.014	0.522	0.699	0.904
NM_001243510.2	0.000	-	-	-	0.000	-	-	-
NM_001243511.2	0.000	-	-	-	0.000	-	-	-
NM_001243512.1	8.930	-0.565	0.676	0.853	15.590	0.914	0.687	0.899
NM_001243513.1	14.120	-1.153	0.051	0.192	24.859	-0.011	0.988	0.996
NM_001243514.1	23.419	-0.035	0.954	0.983	35.173	1.187	0.102	0.328
NM_001243515.1	13.313	-1.374	0.200	0.447	24.522	-0.179	0.901	0.973
NM_001243518.1	23.787	-0.913	0.058	0.209	68.218	2.014	0.001	0.014
NM_001243519.1	16.355	0.476	0.521	0.754	17.576	0.870	0.516	0.809
NM_001438.3	40.861	-1.694	0.198	0.445	86.538	0.111	0.842	0.955
NM_206594.2	79.812	-0.223	0.620	0.820	108.824	0.483	0.428	0.744
NM_206595.2	15.310	-0.486	0.554	0.775	37.607	2.033	0.230	0.539

FC, Fold change; P Adj, Adjusted p value

Supplementary Figure 8 The alternative splicing of ESRRG in prolactinomas with or without *SF3B1* mutation.

a, The count of U2AF2 binding motifs recognized in the 15 proven alternatively splicing events of 14 genes. **b**, The count of introns containing the U2AF2 binding motifs on the last 100bp at the 3' ends in each transcript of ESRRG. Over half of the annotated introns are

ending with the U2AF2 binding motif, which indicates that the known splicing of ESRRG would be more likely impacted by the U2 snRNP complex. **c**, The first row of the figure represents the distribution of the density of the U2AF2 binding motifs. The density is defined as the proportion of 7-bp-long (i.e. the length of the U2AF2 binding motif) subsequences that can be recognized by the U2AF2 binding motifs to all 7-bp-long subsequences on the corresponding genome region. The enriched binding motifs on the entire gene body of ESRRG indicate that there would be more abnormal unknown splicing events caused by the U2 snRNP complex on ESRRG. The second row is the existence of the U2AF2 binding motif on the last 100bp at the 3' ends of introns. The last few rows are the transcripts annotated in the RefSeq database. **d**, The transcript-level differential expression analysis of cryptic ESRRG from RNA-Seq data (Normal pituitary n=6, Mutant tumor n=2 and Wild type tumor n=12). Results are given as mean ± SD. The p-values by one-way ANOVA followed by Tukey's multiple comparisons post hoc test are indicated. **e**, The intergroup expression levels of the cryptic ESRRG transcript (NM_001243518.1). ESRRG expression from RNA-seq data in normal pituitaries, prolactinoma samples with mutant or wild type *SF3B1*. Source data are provided as a Source Data file.

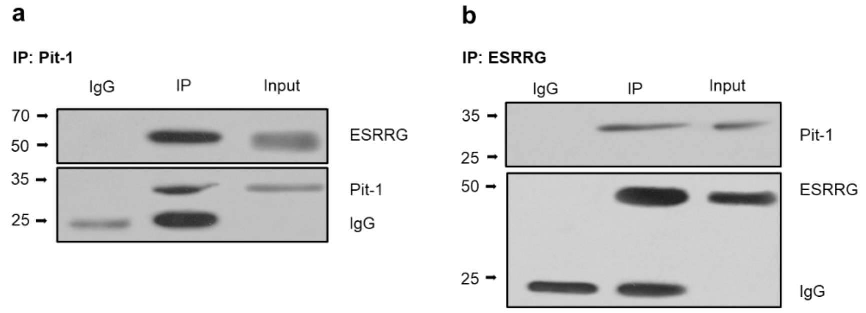

Supplementary Figure 9 Interaction of Pit-1 and ESRRG in prolactinomas.

a, ESRRG specific antibody recognized the complex immunoprecipitated by Pit-1 specific antibody but did not recognize normal IgG. **b**, The Pit-1 specific antibody recognized the complex immunoprecipitated by ESRRG specific antibody but did not recognize normal IgG.

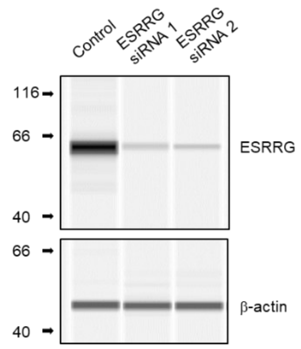

Supplementary Figure 10 Representative western blot for ESRRG expression levels in primary human prolactinoma cells transfected with control or specific ESRRG siRNA. β-actin was used as an internal control.

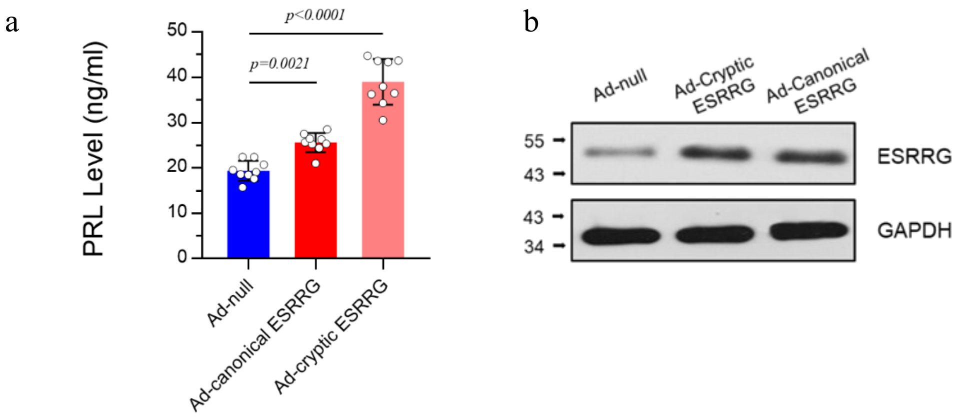

Supplementary Figure 11 Representative PRL secretion (a, n=9 per group) and western blot (b) for ESRRG expression levels in primary human prolactinoma cells transfected with Ad-null, Ad- canonical ESRRG and cryptic ESRRG are shown respectively. Results are expressed as mean ± SD. The p-values by one-way ANOVA followed by Tukey's multiple comparisons post hoc test are indicated. Source data are provided as a Source Data file.

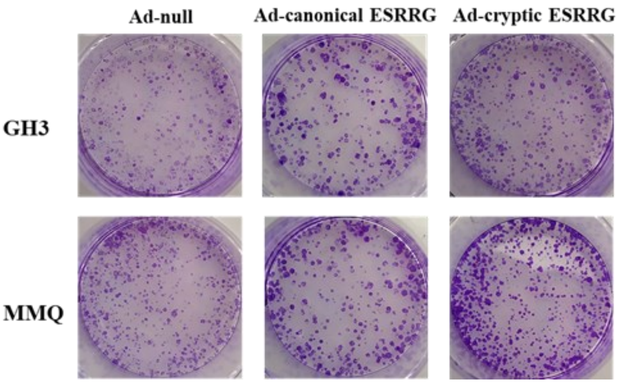

Supplementary Figure 12 Focus formations assay.

The focus formation of GH3/MMQ cells transduced with Ad-null, Ad-canonical ESRRG and Ad-cryptic ESRRG.

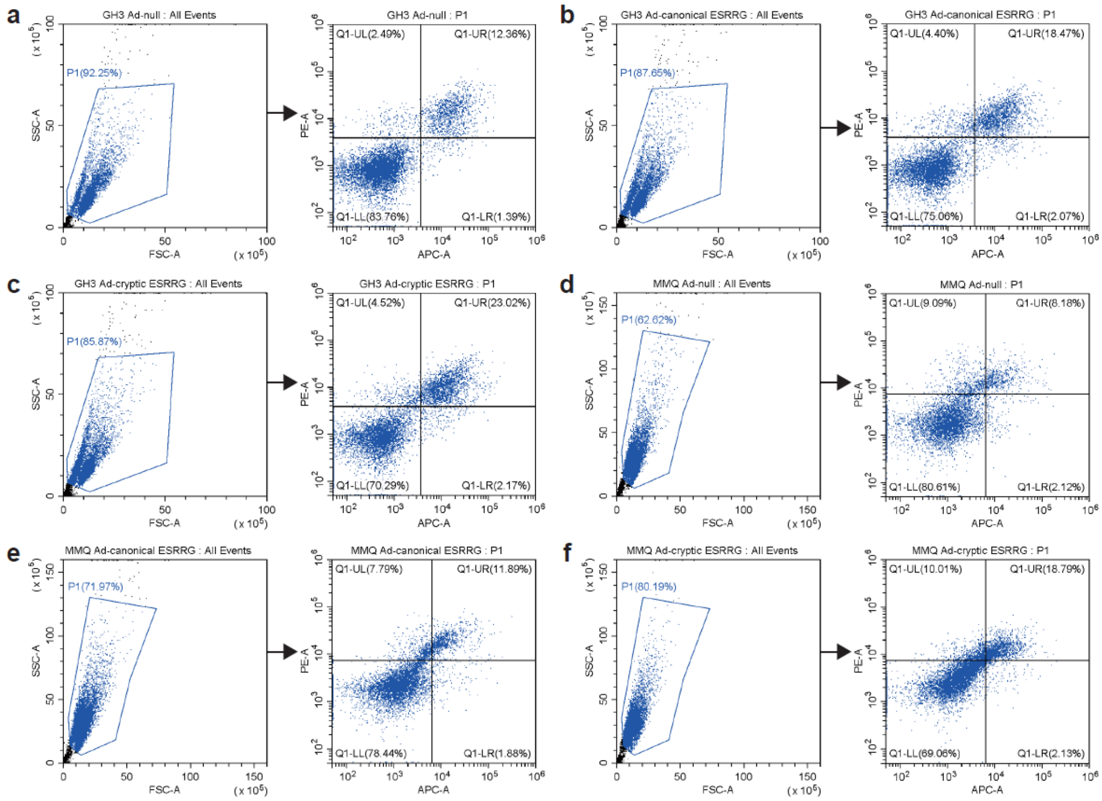

Supplementary Figure 13 The gating strategy for flow cytometry of Fig. 6g, h. Annexin V/PI staining and flow cytometry showed the percentages of apoptosis of GH3/MMQ cells transduced with Ad-null, Ad-canonical ESRRG and Ad-cryptic ESRRG. a-c, GH3 cells. d-f, MMQ cells.

Supplementary Table 1 Overview on the patients included in this study and summary of *SF3B1*^{R625H} mutational status of these patients

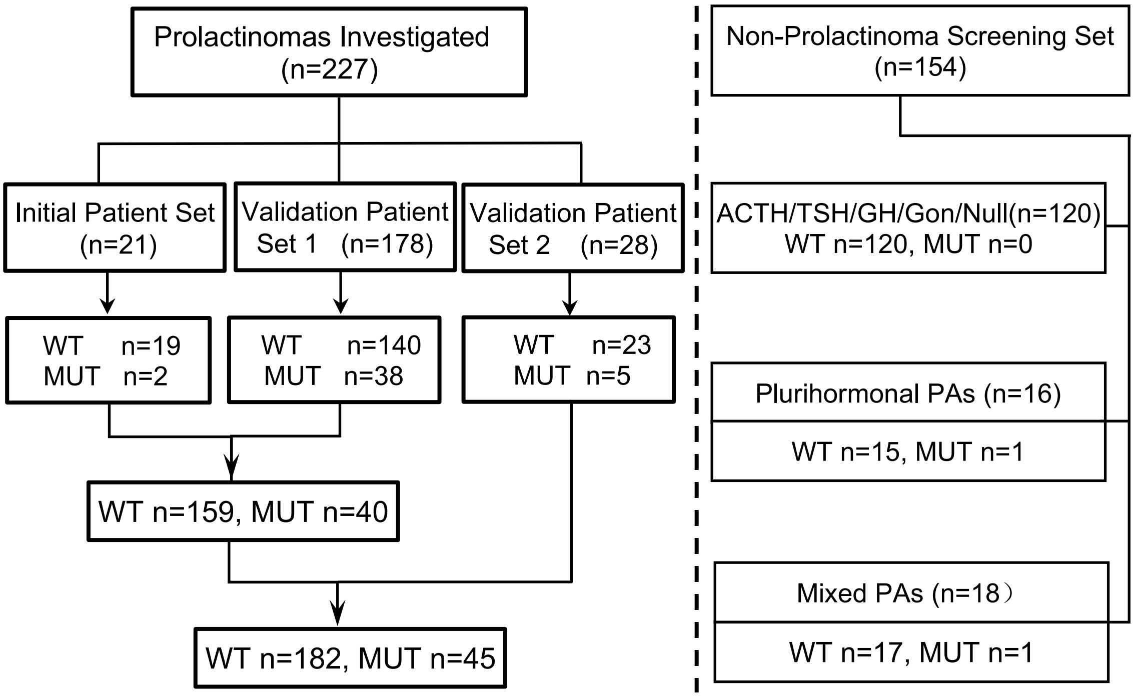

PA, Pituitary adenoma; WT, Wild type; MUT, Mutant; GH, Somatotroph adenoma; ACTH, Corticotroph adenoma; TSH, Thyrotroph adenoma; Gon, Gonadotroph adenoma; Null, Null cell adenoma; Mixed PAs, Mammosomatotroph/mixed somatotroph and lactotroph adenoma.

Supplementary Table 2 The mutations found in 21 patients with prolactinoma by whole genome sequencing (confirmed by Sanger sequencing or Time of Flight Mass Spectrometer (TOF)).

Gene	Sample	Chr	Position	Ref	Alt	Mutation Type	Transcript	Exon	CDS Level	Protein Level
ACPP	01P020	3	132051078	A	G	Missense	NM_001099	exon4	c.G346A	p.A116T
ACTL6B	02P016	7	100253445	T	G	Missense	NM_016188	exon2	c.C83A	p.A28D
ADAMTS4	01P020	1	161168182	A	G	Missense	NM_005099	exon1	c.C236T	p.A79V
AGTR1	01P020	3	148459581	A	G	Nonsense	NM_009585	exon2	c.G759A	p.W253X
ALPK2	01P017	18	56246261	A	T	Missense	NM_052947	exon4	c.A1747T	p.T583S
CCDC65	01P020	12	49312558	C	G	Missense	NM_001286957	exon6	c.G469C	p.V157L
CENPN	01P020	16	81061783	C	T	Missense	NM_001270473	exon9	c.C758T	p.T253M
DHX33	02P022	17	5372124	A	G	Missense	NM_020162	exon1	c.C56T	p.P19L
DNAH6	02P015	2	84913611	A	T	Missense	NM_001370	exon44	c.A7067T	p.K2356I
DOPEY1	01P020	6	83869655	C	G	Missense	NM_001199942	exon37	c.C6911G	p.S2304C
EXOC8	01P019	1	231471445	C	T	Missense	NM_175876	exon1	c.G2047A	p.E683K
FAM162A	01P020	3	122121654	A	C	Missense	NM_014367	exon2	c.C82A	p.L28I
GALNT9	01P020	12	132862879	A	G	Missense	NM_001122636	exon2	c.C376T	p.R126C
GRB2	02P023	17	73322052	C	T	Missense	NM_002086	exon4	c.A226G	p.K76E
HSPA4L	01P019	4	128725218	A	T	Missense	NM_014278	exon8	c.T961A	p.L321I
INCENP	01P017	11	61897801	A	G	Missense	NM_001040694	exon4	c.G802A	p.D268N
LOXL4	01P020	10	100017852	A	G	Nonsense	NM_032211	exon7	c.C991T	p.Q331X
MCM3AP	02P016	21	47664916	C	T	Missense	NM_003906	exon23	c.G4843A	p.V1615M
MPC2	01P019	1	167893758	A	C	Missense	NM_001143674	exon3	c.T127G	p.F43V
MRC2	01P020	17	60757231	T	G	Missense	NM_006039	exon14	c.G2266T	p.G756C
MTMR12	01P020	5	32312869	A	G	Missense	NM_001040446	exon1	c.C76T	p.P26S
MYO9A	01P020	15	72141257	A	T	Nonsense	NM_006901	exon39	c.A6766T	p.K2256X
ORAI2	01P027	7	102079487	C	G	Missense	NM_032831	exon2	c.C84G	p.D28E
PDE4C	01P020	19	18331123	A	G	Missense	NM_001098818	exon7	c.C619T	p.R207W
PDHX	02P023	11	34938233	C	T	Missense	NM_001166158	exon1	c.C31T	p.P11S
PDPR	01P019	16	70154480	A	G	Missense	NM_017990	exon3	c.A85G	p.T29A
PHLPP1	01P020	18	60383748	T	G	Missense	NM_194449	exon1	c.G832T	p.D278Y

PRRC2C	01P017	1	171514665	A	G	Missense	NM_015172	exon17	c.A4805G	p.N1602S
PZP	01P027	12	9317778	A	G	Missense	NM_002864	exon19	c.C2444T	p.T815I
RIPK4	01P020	21	43161434	A	G	Missense	NM_020639	exon8	c.C1919T	p.P640L
RNF20	02P023	9	104323499	C	T	Missense	NM_019592	exon18	c.T2636C	p.F879S
RYR3	01P020	15	33873770	A	G	Missense	NM_001036	exon14	c.A1499G	p.H500R
SATB2	02P015	2	200298216	A	C	Missense	NM_001172509	exon3	c.G191T	p.C64F
SF3B1	01P019	2	198267483	C	T	Missense	NM_012433	exon14	c.G1874A	p.R625H
SF3B1	01P020	2	198267483	C	T	Missense	NM_012433	exon14	c.G1874A	p.R625H
SH2D3C	02P015	9	130502648	C	T	Missense	NM_001142533	exon8	c.A1615G	p.I539V
SREBF2	01P020	22	42264687	A	T	Missense	NM_004599	exon3	c.A611T	p.Q204L
THBD	02P015	20	23029341	C	G	Missense	NM_000361	exon1	c.C801G	p.C267W
TRIM35	01P020	8	27146786	A	G	Missense	NM_171982	exon5	c.C797T	p.T266I
UBR2	02P015	6	42641573	T	G	Missense	NM_015255	exon37	c.G4131T	p.W1377C
ZNF695	01P028	1	247150876	C	T	Missense	NM_020394	exon4	c.A941G	p.H314R
ZNF804B	02P015	7	88962838	A	T	Missense	NM_181646	exon4	c.A542T	p.Q181L
ACAP3	01P004	1	1233201	C	T	Splice Site	NM_030649	exon15	c.1128+1G>A	
ADRM1	01P028	20	60883079	G	A	Missense	NM_001281437	exon7	c.G742A	p.D248N
C19orf22	01P006	19	900919	C	A	Nonsense	NM_138774	exon4	c.G385T	p.E129X
CARM1	01P006	19	11031545	C	A	Missense	NM_199141	exon13	c.C1460A	p.S487Y
CCDC42B	01P002	12	113590663	T	A	Missense	NM_001144872	exon3	c.T238A	p.S80T
CDH1	01P005	16	68867194	A	G	Missense	NM_004360	exon16	c.A2441G	p.N814S
CHI3L2	01P001	1	111777638	C	A	Missense	NM_001025197	exon4	c.C409A	p.P137T
DHRS7C	01P008	17	9674885	T	G	Missense	NM_001105571	exon6	c.C856A	p.R286S
DMXL2	01P004	15	51763600	T	C	Splice Site	NM_001174116	exon30	c.7214-2A>G	
DOPEY1	01P020	6	83869655	C	G	Missense	NM_001199942	exon37	c.C6911G	p.S2304C
DYSF	01P006	2	71839775	T	C	Missense	NM_001130976	exon38	c.T4130C	p.L1377P
ECE1	01P005	1	21616613	C	T	Missense	NM_001113349	exon1	c.A80C	p.D27A
ECE1	01P005	1	21616612	T	G	Missense	NM_001113349	exon1	c.G79A	p.D27N
ETFB	01P003	19	51856529	C	T	Missense	NM_001014763	exon2	c.G505A	p.A169T
EXOC8	01P019	1	231471445	C	T	Missense	NM_175876	exon1	c.G2047A	p.E683K

FAM177B	01P006	1	222919978	G	A	Missense	NM_207468	exon3	c.G91A	p.G31R
G6PC2	01P017	2	169764363	A	G	Missense	NM_021176	exon5	c.G842A	p.S281N
GMNC	01P008	3	190573337	T	G	Missense	NM_001146686	exon5	c.C752A	p.T251N
HPSE2	02P015	10	100904142	C	T	Missense	NM_001166244	exon3	c.G463A	p.D155N
HYDIN	01P008	16	71022295	C	T	Splice Site	NM_001270974	exon27	c.3984+1G>A	
KIF2B	02P023	17	51900887	C	T	Missense	NM_032559	exon1	c.C493T	p.R165W
MDN1	01P005	6	90368513	C	G	Missense	NM_014611	exon89	c.G14837C	p.S4946T
MECOM	01P009	3	168812986	C	T	Missense	NM_001163999	exon11	c.G2309A	p.R770K
MPDZ	01P003	9	13140147	G	A	Missense	NM_001261406	exon27	c.C3743T	p.A1248V
NOTCH2NL	01P003	1	145248895	G	A	Splice Site	NM_203458	exon2	c.38+1G>A	
NUP54	01P017	4	77051886	C	T	Missense	NM_001278603	exon7	c.A835G	p.M279V
OR5AN1	01P003	11	59132719	C	T	Missense	NM_001004729	exon1	c.C788T	p.S263F
ORAI2	01P027	7	102079487	C	G	Missense	NM_032831	exon2	c.C84G	p.D28E
PAQR5	01P012	15	69692338	A	C	Missense	NM_001104554	exon8	c.C635A	p.A212E
PCNXL2	01P009	1	233394269	C	T	Missense	NM_014801	exon5	c.G1339A	p.E447K
PITHD1	01P017	1	24105953	A	G	Missense	NM_020362	exon2	c.A224G	p.E75G
RBL1	02P023	20	35689594	A	C	Missense	NM_002895	exon9	c.G1162T	p.A388S
SACS	01P003	13	23905003	G	A	Missense	NM_001278055	exon8	c.C12571T	p.P4191S
SPAG9	01P008	17	49082626	C	T	Splice Site	NM_001130527	exon12	c.1383-2A>G	
SREBF2	01P020	22	42264687	A	T	Missense	NM_004599	exon3	c.A611T	p.Q204L
STIP1	02P023	11	63964765	A	G	Missense	NM_001282652	exon6	c.G836A	p.G279E
TERF2	01P027	16	69419359	C	T	Missense	NM_005652	exon2	c.A410G	p.H137R
THBD	02P015	20	23029341	G	C	Missense	NM_000361	exon1	c.C801G	p.C267W
TUSC5	01P006	17	1198818	G	A	Missense	NM_172367	exon2	c.G421A	p.G141S
UNC13C	01P012	15	54306248	A	C	Missense	NM_001080534	exon1	c.C1148A	p.T383N
VCX	01P012	X	7811584	C	T	Missense	NM_013452	exon3	c.C148T	p.R50C
WDFY3	01P001	4	85674885	C	T	Missense	NM_014991	exon35	c.G5704A	p.V1902I
YARS	02P015	1	33263369	T	C	Missense	NM_003680	exon5	c.G586A	p.E196K
YTHDC1	01P009	4	69195940	A	C	Missense	NM_133370	exon7	c.G1157T	p.R386I
ZDBF2	01P002	2	207173825	G	T	Missense	NM_020923	exon5	c.G4573T	p.V1525L

ZNF407	01P019	18	72343166	T	A	Missense	NM_001146189	exon1	c.T191A	p.I64K
ZNF687	01P027	1	151259759	G	A	Missense	NM_020832	exon2	c.G992A	p.R331Q
ZYX	01P003	7	143078867	C	T	Missense	NM_001010972	exon2	c.C203T	p.P68L

Chr, Chromosome; Ref, Reference; Alt, Alteration; CDS, Coding sequence

Supplementary Table 3 The summary of somatic variants of *SF3B1*^{R625H} detected by digital PCR in pituitary adenoma samples.

Patient	WT	Mut	FA (%)	WT	Mut	FA (%)	WT	Mut	FA (%)	WT	Mut	FA (%)	Tumor Type
**01P019**	255	194	76.08	575	481	83.65	994	592	59.56	927	588	63.43	PRL
	885	603	68.14	195	190	97.44	172	111	64.53	86	68	79.07	
	44	26	59.09	35	14	40	311	205	65.92	1467	2327	158.62	
	291	158	54.3	1454	1933	132.94	295	167	56.61	347	176	50.72	
	751	463	61.65	305	163	53.44	271	160	59.04	305	153	50.16	
	259	163	62.93	885	526	59.44	222	125	56.31				
**01P020**	236	119	50.42	1194	441	36.93	911	394	43.25	1035	474	45.8	PRL
	800	252	31.5	69	34	49.28	153	78	50.98	76	41	53.95	
	34	13	38.24	20	8	40	324	139	42.9	77	34	44.16	
	239	80	33.47	294	126	42.86	316	120	37.97	282	129	45.74	
	214	104	48.6	308	129	41.88	847	363	42.86	471	228	48.41	
	228	96	42.11	222	100	45.05	237	116	48.95	288	90	31.25	
	298	101	33.89	635	352	55.43	233	108	46.35	291	131	45.02	
**02P157**	57	3	5.26	23	0	0	21	1	4.76	68	1	1.47	PRL
**02P161**	52	3	5.77	59	46	77.97	98	4	4.08				PRL
**02P163**	50	1	2	40	0	0	53	1	1.89	51	1	1.96	PRL
**02P193**	489	53	10.84	1039	5	0.48	155	18	11.61	165	58	35.15	PRL
	169	24	14.2										
**02P242**	124	1	0.81	246	8	3.25	250	4	1.6				PRL
**02P249**	87	5	5.75	31	0	0	24	1	4.17	21	1	4.76	PRL
	45	2	4.44										
**01P043**	32	0	0	40	1	2.5	54	2	3.7	72	1	1.39	PRL
	59	1	1.69										
**01P059**	138	0	0	32	1	3.13	156	2	1.28	87	2	2.3	PRL
	50	1	2										
**01P060**	30	6	20	57	0	0	175	4	2.29	217	2	0.92	PRL
**01P061**	21	1	4.76	65	1	1.54	42	1	2.38				PRL
**01P063**	453	6	1.32	411	5	1.22	648	6	0.93				PRL
**01P074**	310	18	5.81	387	47	12.14	266	31	11.65	276	40	14.49	PRL
**01P076**	358	4	1.12	205	1	0.49	364	2	0.55	105	1	0.95	PRL
	134	1	0.75										
**01P083**	213	4	1.88	75	3	4	196	2	1.02				PRL
**01P084**	230	3	1.3	185	3	1.62	289	2	0.69				PRL
**01P086**	135	3	2.22	88	2	2.27	116	2	1.72				PRL
**07P090**	327	4	1.22	39	1	2.56	256	4	1.56				PRL
**01P091**	285	5	1.75	88	2	2.27	306	7	2.29				PRL
**01P099**	96	1	1.04	91	43	47.25	99	1	1.01	352	4	1.14	PRL
	152	1	0.66										

**01P100**	292	4	1.37	289	3	1.04	712	2	0.28	382	3	0.79	PRL
**01P107**	77	0	0	113	2	1.77	240	3	1.25	97	3	3.09	PRL
	133	2	1.5										
**01P109**	166	3	1.81	131	1	0.76	214	1	0.47	251	2	0.8	PRL
**01P110**	217	3	1.38	190	2	1.05	310	4	1.29				PRL
**01P125**	20	1	5	20	0	0	59	1	1.69	52	2	3.85	PRL
	35	1	2.86										
**01P126**	92	1	1.09	123	2	1.63	135	2	1.48				PRL
**01P129**	179	6	3.35	209	6	2.87	220	3	1.36	216	2	0.93	PRL
**01P040**	372	6	1.61	379	4	1.06	610	2	0.33	58	2	3.45	PRL
**01P137**	503	6	1.19	522	1	0.19	163	1	0.61	146	2	1.37	PRL
**01P141**	222	5	2.25	168	2	1.19	244	3	1.23	423	8	1.89	PRL
**01P162**	36	2	5.56	40	1	2.5	44	1	2.27	44	1	2.27	PRL
**01P174**	397	4	1.01	348	3	0.86	285	3	1.05				PRL
**01P178**	91	2	2.2	75	1	1.33	327	1	0.31	166	2	1.2	PRL
**01P188**	118	3	2.54	32	0	0	405	4	0.99	120	2	1.67	PRL
**01P190**	50	4	8	67	1	1.49	58	1	1.72	201	4	1.99	PRL
	100	3	3										
**01P195**	181	4	2.21	201	5	2.49	340	3	0.88				PRL
**01P197**	111	3	2.7	126	2	1.59	222	2	0.9				PRL
**01P232**	123	4	3.25	139	2	1.44	213	3	1.41				PRL
**01P237**	358	3	0.84	387	5	1.29	522	3	0.57				PRL
**03P004**	140	5	3.57	70	1	1.43	26	1	3.85	72	1	1.39	PRL
	60	2	3.33	203	6	2.96	248	4	1.61	285	2	0.7	
**03P005**	310	2	0.65	271	2	0.74	347	2	0.58	234	0	0	PRL
	394	2	0.51	295	3	1.02	841	5	0.59				
**03P002**	371	3	0.81	505	2	0.4	132	1	0.76	116	1	0.86	PRL
	98	2	2.04	87	2	2.3							
**03P001**	164	4	2.44	191	0	0	128	5	3.91	127	1	0.79	PRL
	120	2	1.67										
**03P003**	465	5	1.08	321	1	0.31	416	4	0.96	377	5	1.33	PRL
**01M031**	114	3	2.63	26	1	3.85	25	0	0	80	1	1.25	Mixed *
**02M209**	217	3	1.38	309	5	1.62	85	1	1.18				Pluri#

All positive result (fractional abundance ≥0.5%) were identified more than 3 times and the average value was presented.

FA, Fractional Abundance, it was the ratio of SF3B1R625H reads to wild type reads. The wild type replication <20 copies were excluded.

WT, Positive Events of wild type of *SF3B1*; Mut, Positive Events of mutant *SF3B1*^{R625H}

* Mixed, Mammosomatotroph/mixed somatotroph and lactotroph adenoma. The patient had symptoms of acromegaly with hyperprolactinemia (PRL 250ng/ml) and high GH level (16.7ng/ml). The tumor had positive immunostaining for PRL and GH.

# Pluri: Plurihormonal adenoma. It was a non-functioning pituitary adenoma with positive immunostaining for PRL, TSH and ACTH.

PRL, Prolactinoma

Supplementary Table 4 The list of significant alternative splicing events in the comparison of *SF3B1*^{R625H} with *SF3B1*^{WT}. The p values by rMATS (replicate Multivariate Analysis of Transcript Splicing) are indicated.

a, A3SS splicing type

Type	Gene	Chr	Strand	Long ES	Long EE	Short ES	Short EE	Flanking ES	Flanking EE	P Value	FDR	Inc Level Difference
A3SS	*ANKHD1*	5	+	139818045	139818202	139818078	139818202	139815688	139815842	0	0	-0.697
A3SS	*MTERF2*	12	-	107378892	107379003	107378892	107378993	107380746	107380944	0	0	-0.814
A3SS	*DYNLL1*	12	+	120934204	120934356	120934218	120934356	120933858	120934019	0	0	0.509
A3SS	*ORAI2*	7	+	102076648	102076780	102076671	102076780	102073976	102074108	0	0	0.818
A3SS	*BCLAF1*	6	-	136596980	136597646	136596980	136597127	136599002	136599914	0	0	-0.314
A3SS	*TMEM14C*	6	+	10724788	10724866	10724802	10724866	10723147	10723474	0	0	0.678
A3SS	*MED6*	14	-	71059596	71059726	71059596	71059705	71060012	71060095	1.11E-16	3.93E-14	0.411
A3SS	*FAM208A*	3	-	56654159	56657871	56654159	56657594	56658506	56658671	2.00E-15	6.19E-13	-0.28
A3SS	*VAMP1*	12	-	6571403	6573652	6571403	6572008	6574055	6574107	3.00E-15	7.98E-13	-0.2
A3SS	*VAMP1*	12	-	6571403	6572135	6571403	6572008	6574055	6574107	3.22E-15	7.98E-13	-0.203
A3SS	*MORF4L2*	X	-	102933426	102933579	102933426	102933528	102940098	102940188	2.65E-14	5.98E-12	-0.243
A3SS	*NCOA7*	6	+	126210084	126211127	126210117	126211127	126206304	126206489	3.19E-13	6.58E-11	0.312
A3SS	*SERBP1*	1	-	67890570	67890660	67890570	67890642	67890765	67890906	2.87E-11	5.47E-09	0.264
A3SS	*CDC27*	17	-	45229171	45229302	45229171	45229284	45232037	45232152	1.32E-10	2.33E-08	0.273
A3SS	*ZBED5*	11	-	10874250	10876665	10874250	10876633	10877690	10877804	1.26E-08	2.08E-06	0.327
A3SS	*APBB3*	5	-	139941171	139941292	139941171	139941286	139941684	139941812	3.21E-08	4.97E-06	-0.863
A3SS	*THRA*	17	+	38249272	38250120	38249389	38250120	38245458	38245586	5.32E-08	7.76E-06	-0.298
A3SS	*VEZT*	12	+	95663814	95663964	95663826	95663964	95660132	95660408	1.51E-07	2.08E-05	0.252

A3SS	*ESRRG*	1	-	216737560	216737743	216737560	216737722	216741329	216741440	4.42E-07	5.77E-05	0.434
A3SS	*YY1AP1*	1	-	155638417	155638568	155638417	155638508	155640110	155640255	5.97E-07	7.40E-05	0.211
A3SS	*HSF4*	16	+	67201363	67201502	67201377	67201502	67201022	67201125	2.10E-06	0.00024735	0.513
A3SS	*CREM*	10	+	35500181	35501886	35500583	35501886	35495822	35495979	2.71E-06	0.00030488	-0.268
A3SS	*CHD4*	12	-	6711125	6711341	6711125	6711320	6711541	6711663	7.97E-06	0.00085815	0.192
A3SS	*GBGT1*	9	-	136028334	136029648	136028334	136029629	136030564	136030699	9.12E-06	0.00094131	-0.397
A3SS	*GORASP1*	3	-	39141795	39141994	39141795	39141945	39142237	39142368	2.72E-05	0.00263337	-0.272
A3SS	*RC3H1*	1	-	173912579	173912753	173912579	173912726	173915613	173915746	2.76E-05	0.00263337	0.225
A3SS	*TRIM26*	6	-	30166442	30167041	30166442	30166930	30181081	30181271	4.61E-05	0.00423208	-0.107
A3SS	*ZSWIM8*	10	+	75554298	75554397	75554313	75554397	75553904	75554088	6.71E-05	0.00593457	0.267
A3SS	*ZNF142*	2	-	219523702	219523853	219523702	219523758	219524091	219524263	0.00018475	0.01578034	0.256
A3SS	*MVP*	16	+	29841794	29841995	29841835	29841995	29831714	29831831	0.00021676	0.01789703	0.391
A3SS	*MAP2K7*	19	+	7976299	7976463	7976320	7976463	7976134	7976215	0.00024413	0.01950679	0.202
A3SS	*SLC25A27*	6	+	46644119	46645927	46644383	46645927	46638862	46638965	0.00030371	0.02287136	-0.21
A3SS	*SCNM1*	1	+	151140619	151140814	151140669	151140814	151139801	151139890	0.00030471	0.02287136	-0.276
A3SS	*GAS8*	16	+	90097583	90097904	90097706	90097904	90094043	90094130	0.00033339	0.02428854	0.43
A3SS	*NEURL2*	20	-	44517110	44517526	44517110	44517512	44518888	44519926	0.00047072	0.03331331	0.519

b, A5SS splicing type

Type	Gene	Chr	Strand	Long ES	Long EE	Short ES	Short EE	Flanking ES	Flanking EE	P Value	FDR	Inc Level Difference
A5SS	*NCOR2*	12	-	124811954	124812179	124812092	124812179	124810736	124810916	9.50E-14	1.00E-10	-0.321
A5SS	*NDUFS5*	1	+	39491966	39492074	39491966	39492070	39494394	39494612	8.20E-14	1.00E-10	-0.254

c, MXE splicing type

Type	Gene	Chr	Strand	1st ES	1st EE	2nd ES	2nd EE	Upstream ES	Upstream EE	Downstream ES	Downstream EE	P Value	FDR	Inc Level Difference
MXE	*TMEM14C*	6	+	10724788	10724866	10725193	10725270	10723147	10723474	10726139	10726241	0	0	0.574
MXE	*H2AFY*	5	-	134686512	134686603	134688635	134688735	134681657	134681747	134696186	134696297	8.22E-14	4.83E-10	0.179
MXE	*TGFBR1*	9	+	101894790	101895021	101900140	101900371	101891136	101891382	101904817	101904985	1.69E-12	6.61E-09	-0.261
MXE	*ORAI2*	7	+	102076671	102076780	102079390	102079628	102073976	102074108	102086959	102097268	1.86E-09	5.46E-06	-0.244
MXE	*STAG3L5P-PVRIG2P-PILRB*	7	+	99952765	99952863	99954372	99954506	99950995	99951635	99955842	99955989	1.83E-08	4.32E-05	0.313
MXE	*LSR*	19	+	35749847	35749967	35753448	35753595	35741217	35741562	35757261	35757435	2.89E-07	0.0005022	0.118
MXE	*SNAP25*	20	+	10273529	10273647	10273808	10273926	10265371	10265420	10277572	10277698	2.99E-07	0.0005022	-0.273
MXE	*CDC27*	17	-	45221248	45221348	45229171	45229302	45219594	45219802	45232037	45232152	4.54E-07	0.0006684	0.151
MXE	*ARHGAP44*	17	+	12877405	12877627	12883374	12883550	12862033	12862214	12887847	12888225	5.12E-07	0.0006702	-0.184
MXE	*ZNF227*	19	+	44721943	44722005	44732598	44732786	44717242	44717397	44738854	44741421	7.94E-07	0.0009343	-0.349
MXE	*SNAP25*	20	+	10273529	10273647	10273808	10273926	10258332	10258374	10277572	10277698	9.38E-07	0.0010035	-0.26
MXE	*ZNF227*	19	+	44721943	44722005	44732598	44732725	44717239	44717397	44738854	44741421	1.21E-06	0.0011853	-0.386
MXE	*SNRPN*	15	+	25207260	25207356	25213078	25213229	25200134	25200209	25219457	25219603	1.41E-06	0.001275	0.159
MXE	*ARHGAP44*	17	+	12883374	12883550	12887847	12888225	12862033	12862214	12893348	12894960	2.81E-06	0.0023631	0.437
MXE	*SNRPN*	15	+	25219457	25219603	25221451	25221563	25213078	25213229	25222023	25222176	3.11E-06	0.0024394	-0.175
MXE	*SNAP25*	20	+	10273529	10273647	10273808	10273926	10265371	10265420	10279915	10280060	1.37E-05	0.010087	-0.296
MXE	*LOC103091866*	1	+	149577607	149577662	149670208	149670452	149576261	149576700	149672300	149672983	2.64E-05	0.0177955	-0.219
MXE	*TMEM14C*	6	+	10724802	10724866	10725193	10725270	10723147	10723474	10726139	10726241	2.72E-05	0.0177955	-0.262

MXE	*BPTF*	17	+	65962687	65962772	65971887	65972074	65960327	65960520	65978367	65980494	3.19E-05	0.0197784	0.175
MXE	*STAU2*	8	-	74585341	74585477	74600888	74601048	74529526	74529686	74621266	74621397	4.74E-05	0.0279004	0.186
MXE	*TMEM14C*	6	+	10725193	10725270	10726139	10726241	10724802	10724866	10728872	10728960	5.49E-05	0.0307848	0.198
MXE	*NEK1*	4	-	170458959	170459062	170476870	170477002	170429919	170430003	170477082	170477246	8.01E-05	0.0428321	-0.298

d, RI splicing type

Type	Gene	Chr	Strand	RI ES	RI EE	Upstream ES	Upstream EE	Downstream ES	Downstream EE	P Value	FDR	Inc Level Difference
**RI**	*TCEB2*	16	-	2821414	2822103	2821414	2821605	2821994	2822103	1.42E-08	9.89E-06	-0.474
**RI**	*NAPA*	19	-	47990890	47991646	47990890	47991147	47991491	47991646	4.17E-08	1.45E-05	-0.245
**RI**	*CUTA*	6	-	33385257	33386065	33385257	33385472	33385645	33386065	7.19E-07	0.00012659	-0.219
**RI**	*HMGN3*	6	-	79910961	79912106	79910961	79911443	79911992	79912106	7.28E-07	0.00012659	-0.171
**RI**	*MAP3K12*	12	-	53880730	53881212	53880730	53880939	53881038	53881212	1.51E-06	0.00020987	-0.404
**RI**	*MAP7D1*	1	+	36641799	36642182	36641799	36641922	36642018	36642182	7.20E-06	0.00083538	-0.245
**RI**	*CIART*	1	+	150254942	150256043	150254942	150255028	150255871	150256043	1.46E-05	0.00144945	-0.317
**RI**	*NFATC4*	14	+	24845499	24848811	24845499	24846084	24846843	24848811	2.37E-05	0.00206506	-0.404
**RI**	*LARP1B*	4	+	129028293	129030059	129028293	129028468	129029037	129030059	0.00011127	0.00774444	-0.244
**RI**	*EFHC1*	6	+	52317485	52319085	52317485	52317635	52318892	52319085	0.00010031	0.00774444	-0.236
**RI**	*RAB24*	5	-	176730172	176730745	176730172	176730321	176730407	176730745	0.00018951	0.01199063	-0.18
**RI**	*FAM195B*	17	-	79780236	79781187	79780236	79780783	79781084	79781187	0.00059773	0.03200163	-0.216
**RI**	*AP1G2*	14	-	24035024	24035369	24035024	24035101	24035272	24035369	0.00057925	0.03200163	-0.271
**RI**	*LUC7L3*	17	+	48827861	48830072	48827861	48828055	48829560	48830072	0.00074453	0.03701368	-0.195
**RI**	*SCRN2*	17	-	45915046	45915816	45915046	45915368	45915635	45915816	0.00089414	0.03929183	-0.195

**RI**	*LRSAM1*	9	+	130213764	130214363	130213764	130213948	130214207	130214363	0.00090326	0.03929183	-0.222
**RI**	*EED*	11	+	85988959	85989785	85988959	85989033	85989440	85989785	0.0011939	0.04887982	-0.205

e, SE splicing type

Type	Gene	Chr	Strand	ES	EE	Upstream ES	Upstream EE	Downstream ES	Downstream EE	P Value	FDR	Inc Level Difference
SE	*LRP8*	1	-	53742363	53742750	53741302	53741425	53755238	53755361	3.96E-14	9.78E-10	0.025
SE	*PLEKHB2*	2	+	131890474	131890588	131884256	131884359	131897742	131897848	5.16E-14	9.78E-10	-0.164
SE	*GLS2*	12	-	56873563	56873685	56871966	56872046	56881720	56882198	5.96E-14	9.78E-10	0.038
SE	*PLCXD1*	X	+	198148	198351	192990	193061	200833	200981	9.63E-14	1.18E-09	-0.141
SE	*VPS13C*	15	-	62315978	62316044	62315609	62315719	62325639	62325741	7.84E-13	7.71E-09	-0.336
SE	*DCAF16*	4	-	17806729	17806848	17802277	17806394	17812069	17812381	3.75E-11	3.07E-07	-0.304
SE	*SYNE4*	19	-	36497651	36497846	36497324	36497573	36499118	36499269	8.15E-11	5.73E-07	0.089
SE	*IFNAR1*	21	+	34715840	34715982	34713304	34713480	34721396	34721596	4.68E-10	2.62E-06	-0.005
SE	*PHF20L1*	8	+	133811340	133811418	133811017	133811106	133816063	133816277	4.79E-10	2.62E-06	0.279
SE	*C21orf62-AS1*	21	+	34160922	34161012	34157107	34157230	34169248	34170016	8.92E-10	4.39E-06	-0.055
SE	*TSTD1*	1	-	161008340	161008463	161007702	161007865	161008669	161008774	1.07E-09	4.77E-06	0.23
SE	*MYL6*	12	+	56554409	56554454	56554026	56554104	56555170	56555366	1.02E-08	3.89E-05	0.198
SE	*SNHG16*	17	+	74555026	74555125	74553845	74553939	74557369	74557484	1.03E-08	3.89E-05	-0.222
SE	*METTL2B*	7	+	128117135	128117227	128116782	128116929	128119211	128119567	1.33E-08	4.67E-05	0.356
SE	*IKBKB*	8	+	42147673	42147791	42129600	42129723	42162704	42162793	1.90E-08	6.11E-05	-0.133
SE	*ABCC5*	3	-	183703091	183703166	183701540	183702682	183705557	183705705	1.99E-08	6.11E-05	0.191
SE	*MYEF2*	15	-	48441228	48441280	48431624	48435268	48441359	48441568	1.53E-07	0.00044254	-0.182

SE	*VPS13C*	15	-	62320556	62320619	62315609	62315719	62325639	62325741	4.81E-07	0.00131532	-0.306
SE	*LSR*	19	+	35753448	35753595	35749847	35749967	35757261	35757435	5.85E-07	0.00151502	-0.224
SE	*SNX19*	11	-	130750516	130750701	130749518	130749606	130773149	130773279	6.68E-07	0.00164388	-0.411
SE	*PHF14*	7	+	11021998	11022786	11014461	11014572	11030329	11030474	7.12E-07	0.00166769	0.195
SE	*PLA2G7*	6	-	46690519	46690662	46684711	46684833	46702916	46703151	1.42E-06	0.00318155	-0.547
SE	*CD44*	11	+	35232792	35232996	35211381	35211612	35236398	35236461	1.59E-06	0.00339536	0.659
SE	*ARHGAP 44*	17	+	12876618	12876636	12862033	12862214	12877405	12877627	4.36E-06	0.00858396	0.251
SE	*TMEM14 C*	6	+	10724788	10724866	10723147	10723474	10726139	10726241	4.30E-06	0.00858396	0.135
SE	*C17orf80*	17	+	71238382	71238490	71231614	71233134	71239034	71239142	6.19E-06	0.01171206	-0.388
SE	*NMRK1*	9	-	77684657	77684729	77683911	77684018	77684810	77684958	6.84E-06	0.0124695	-0.227
SE	*2-Mar*	19	+	8483420	8483691	8478186	8478304	8486672	8486900	8.36E-06	0.01417434	-0.23
SE	*MEG3*	14	+	101302378	101302412	101302072	101302216	101302503	101302637	8.23E-06	0.01417434	0.22
SE	*FAM228B*	2	+	24384375	24384483	24369799	24369956	24387067	24387204	1.12E-05	0.01839426	-0.392
SE	*WDR35*	2	-	20162055	20162088	20160314	20160375	20166484	20166670	1.56E-05	0.02470807	-0.406
SE	*POGZ*	1	-	151413403	151413562	151403141	151403317	151414556	151414681	1.63E-05	0.02499942	-0.175
SE	*EML5*	14	-	89100812	89100833	89093989	89094163	89105152	89105251	1.69E-05	0.0251819	0.01
SE	*RABGEF1*	7	+	66233817	66234012	66205642	66205779	66236869	66237065	2.38E-05	0.03436773	-0.246
SE	*CTNND1*	11	+	57556508	57556627	57529233	57529518	57558856	57559145	2.82E-05	0.03966933	-0.217
SE	*WDPCP*	2	-	63486445	63486544	63401804	63401967	63540382	63540446	3.19E-05	0.04362235	0.045

Chr, Chromosome; ES, Exon Start; EE, Exon End; RI ES, Retained Intron Event (upstream) Exon Start; RI EE, Retained Intron Event (downstream) Exon End; FDR, False Discovery Rate; Inc Level Difference, Inclusion Level Difference (Difference of the longer isoform between SF3B1 mutant and wild type)

Alternative splicing type: A3SS, Alternative 3' splice site; A5SS, Alternative 5' splice site; MXE, Mutually exclusive exons; RI, Retain intron; SE, Skipped exon

Supplementary Table 5 List of the 20 most significant cryptic splicing events in the comparison of *SF3B1*^{R625H} with *SF3B1*^{WT} prolactinomas.

Gene	Type	Chr	Strand	Start Position	End Position	Inc Form Len	Skip Form Len	P Value	FDR	Inc Level Difference
ANKHD1	A3SS	5	+	139815688	139818202	143	117	0	0	-0.697
MTERF2	A3SS	12	-	107380746	107378993	104	94	0	0	-0.814
DYNLL1	A3SS	12	+	120933858	120934356	138	131	0	0	0.509
ORAI2	A3SS	7	+	102073976	102076780	115	92	0	0	0.818
BCLAF1	A3SS	6	-	136599002	136597127	270	135	0	0	-0.314
TMEM14C	A3SS	6	+	10723147	10724866	71	57	0	0	0.678
TMEM14C	MXE	6	+	10723147	10726241	102	100	0	0	0.574
MED6	A3SS	14	-	71060012	71059705	64	43	1.11E-16	3.93E-14	0.411
NCOA7	A3SS	6	+	126206304	126211127	161	135	3.19E-13	6.58E-11	0.312
NCOR2	A5SS	12	-	124810736	124812179	211	80	9.50E-14	1.00E-10	-0.321
VPS13C	SE	15	-	62315609	62325741	46	63	7.84E-13	7.71E-09	-0.336
DCAF16	SE	4	-	17802277	17812381	224	135	3.75E-11	3.07E-07	-0.304
ZBED5	A3SS	11	-	10877690	10876633	132	107	1.26E-08	2.08E-06	0.327
APBB3	A3SS	5	-	139941684	139941286	100	94	3.21E-08	4.97E-06	-0.863
TCEB2	RI	16	-	2821414	2822103	237	102	1.42E-08	9.89E-06	-0.474
STAG3L5P-PVRIG2P-PILRB	MXE	7	+	99950995	99955989	182	254	1.83E-08	4.32E-05	0.313
METTL2B	SE	7	+	128116782	128119567	170	135	1.33E-08	4.67E-05	0.356
ESRRG	A3SS	1	-	216741329	216737722	118	104	4.42E-07	5.77E-05	0.434
MAP3K12	RI	12	-	53880730	53881212	184	135	1.51E-06	0.00020987	-0.404
HSF4	A3SS	16	+	67201022	67201502	93	79	2.10E-06	0.00024735	0.513

Chr, Chromosome; Inc Form Len, Length of the longer isoform (Inclusion isoform); Skip Form Len, Length of the shorter isoform (Skipped isoform); FDR, False Discovery Rate; Inc Level Difference, Inclusion Level Difference (Difference of the longer isoform between *SF3B1* mutant and wild type). The p values by rMATS(replicate Multivariate Analysis of Transcript Splicing) are indicated.

Supplementary Table 6 Inclusion and exclusion criteria.

Inclusion Criteria
● PRL > 90ng ml^-1
● Clinical symptoms related to hyperprolactinemia or mass effect, including amenorrhea, galactorrhea and dysgenesia in female, headache and visual impairment etc.
● Pathological diagnosis was pituitary adenoma and immunohistochemistry showed positive staining for PRL
● MRI/CT showed a sellar region lesion
● Informed consent signed
**Exclusion Criteria**
● Preoperative blood GH level was higher than normal
● Immunohistochemistry showed strong positive staining for GH/ACTH/TSH/FSH/LH
● Not enough DNA of tumor for mutation analysis
● History of pituitary surgery or radiotherapy

PRL, Prolactin; MRI, Magnetic Resonance Imaging; CT, Computed Tomography; GH, Growth Hormone; ACTH, Adrenocorticotropic Hormone; TSH, Thyroid Stimulating Hormone; FSH, Follicle Stimulating Hormone; LH, Luteinizing Hormone.

Supplementary Table 7 The clinical data of 227 prolactinoma patients.

Serial Number	Group	SF3B1^{R625H}	Gender	Age (Years)	PRL (ng/ml)	Tumor Size (cm)	Knosp Classification	Tumor Ivasion	Tumor Progression	PFS (weeks)	PRL/Tumor size	Source of specimens
01P019	Initial Patient Set	Mutation	Male	31	6000	7.6	4	Yes	Yes	31.29	789.47	Beijing Tiantan Hospital
01P020	Initial Patient Set	Mutation	Male	55	5762	4.9	4	Yes	Yes	17.00	1175.92	Beijing Tiantan Hospital
01P013	Initial Patient Set	Wild	Male	20	197	3.3	4	Yes	No	115.14	59.70	Beijing Tiantan Hospital
01P009	Initial Patient Set	Wild	Male	48	3112	3.4	4	Yes	No	188.29	915.29	Beijing Tiantan Hospital
02P015	Initial Patient Set	Wild	Male	65	2560	3.3	4	Yes	No	98.29	775.76	Sanbo Brain Hospital
01P008	Initial Patient Set	Wild	Male	37	464	2	0	No	No	203.14	232.00	Beijing Tiantan Hospital
02P022	Initial Patient Set	Wild	Female	15	314	2.4	2	No	No	54.00	130.83	Sanbo Brain Hospital
01P028	Initial Patient Set	Wild	Female	38	133	3.6	4	Yes	No	46.29	36.94	Beijing Tiantan Hospital
01P012	Initial Patient Set	Wild	Male	45	2071	4.2	2	No	No	128.14	493.10	Beijing Tiantan Hospital
01P004	Initial Patient Set	Wild	Male	43	1068	6.2	4	Yes	No	218.00	172.26	Beijing Tiantan Hospital
01P006	Initial Patient Set	Wild	Female	54	145	1.3	0	No	Yes	47.29	111.54	Beijing Tiantan Hospital
01P001	Initial Patient Set	Wild	Female	25	103	1.4	0	No	No	12.86	73.57	Beijing Tiantan Hospital
01P005	Initial Patient Set	Wild	Female	50	150	1.7	1	No	No	209.86	88.24	Beijing Tiantan Hospital
01P027	Initial Patient Set	Wild	Male	22	509	3.3	2	No	No	13.00	154.24	Beijing Tiantan Hospital
01P011	Initial Patient Set	Wild	Female	48	127	1.4	1	No	No	129.14	90.71	Beijing Tiantan Hospital
01P017	Initial Patient Set	Wild	Female	44	1960	3	2	No	No	71.57	653.33	Beijing Tiantan Hospital
01P003	Initial Patient Set	Wild	Male	42	189	5.4	3	Yes	No	72.29	35.00	Beijing Tiantan Hospital

01P002	Initial Patient Set	Wild	Male	49	1122	2.4	1	No	No	120.14	467.50	Beijing Tiantan Hospital
01P024	Initial Patient Set	Wild	Female	20	766	2.3	3	Yes	No	34.43	333.04	Beijing Tiantan Hospital
02P016	Initial Patient Set	Wild	Male	30	1389	3	3	Yes	No	96.86	463.00	Sanbo Brain Hospital
02P023	Initial Patient Set	Wild	Male	37	2119	6.1	4	Yes	No	50.14	347.38	Sanbo Brain Hospital
02P161	Validation Patient Set 1	Mutation	Male	39	6000	8.9	4	Yes	No	289.29	674.16	Sanbo Brain Hospital
02P193	Validation Patient Set 1	Mutation	Male	20	728.2	4.8	2	Yes	Yes	85.14	151.71	Sanbo Brain Hospital
01P074	Validation Patient Set 1	Mutation	Female	19	223	1.6	1	No	No	413.57	139.38	Beijing Tiantan Hospital
01P099	Validation Patient Set 1	Mutation	Female	21	3404	2	1	No	No	373.57	1702.00	Beijing Tiantan Hospital
01P060	Validation Patient Set 1	Mutation	Male	54	615	2.1	1	No	No	336.71	292.86	Beijing Tiantan Hospital
02P249	Validation Patient Set 1	Mutation	Male	44	2931	5.4	4	Yes	No	95.43	542.78	Sanbo Brain Hospital
01P190	Validation Patient Set 1	Mutation	Male	53	1262	2	1	No	No	68.57	631.00	Beijing Tiantan Hospital
01P162	Validation Patient Set 1	Mutation	Male	20	1802	4.9	2	No	No	287.29	367.76	Beijing Tiantan Hospital
01P061	Validation Patient Set 1	Mutation	Male	41	2655	2.6	2	No	No	432.29	1021.15	Beijing Tiantan Hospital
02P157	Validation Patient Set 1	Mutation	Female	38	131	0.8	0	No	No	62.14	163.75	Sanbo Brain Hospital
01P125	Validation Patient Set 1	Mutation	Male	17	1394	4.8	4	Yes	No	71.29	290.42	Beijing Tiantan Hospital
01P083	Validation Patient Set 1	Mutation	Male	41	1994	3	3	Yes	No	389.86	664.67	Beijing Tiantan Hospital
01P129	Validation Patient Set 1	Mutation	Female	40	226	1	0	No	No	339.57	226.00	Beijing Tiantan Hospital
01P091	Validation Patient Set 1	Mutation	Male	23	513	1.6	2	No	No	370.43	320.63	Beijing Tiantan Hospital
01P086	Validation Patient Set 1	Mutation	Male	44	593	4	2	No	No	389.71	148.25	Beijing Tiantan Hospital
01P232	Validation Patient Set 1	Mutation	Male	47	312	2.8	4	Yes	Yes	5.43	111.43	Beijing Tiantan Hospital
02P242	Validation Patient Set 1	Mutation	Female	35	158	1.2	1	No	No	156.29	131.67	Sanbo Brain Hospital

01P195	Validation Patient Set 1	Mutation	Male	53	1033	2.6	1	No	No	238.29	397.31	Beijing Tiantan Hospital
01P043	Validation Patient Set 1	Mutation	Male	39	3877	4.1	4	Yes	No	472.71	945.61	Beijing Tiantan Hospital
07P090	Validation Patient Set 1	Mutation	Male	26	870	1.9	1	No	No	383.86	457.89	Beijing Tiantan Hospital
01P059	Validation Patient Set 1	Mutation	Male	33	592	1.5	0	No	No	435.43	394.67	Beijing Tiantan Hospital
01P197	Validation Patient Set 1	Mutation	Male	32	5076	4.1	2	No	Yes	14.00	1238.05	Beijing Tiantan Hospital
01P141	Validation Patient Set 1	Mutation	Male	55	632	2.3	1	No	No	314.71	274.78	Beijing Tiantan Hospital
01P040	Validation Patient Set 1	Mutation	Female	47	3929	3.8	4	Yes	Yes	141.43	1033.95	Beijing Tiantan Hospital
01P107	Validation Patient Set 1	Mutation	Male	43	274	2.1	2	No	No	367.29	130.48	Beijing Tiantan Hospital
02P163	Validation Patient Set 1	Mutation	Female	20	217	0.6	0	No	No	284.43	361.67	Sanbo Brain Hospital
01P126	Validation Patient Set 1	Mutation	Male	39	753	2	1	No	No	341.57	376.50	Beijing Tiantan Hospital
01P188	Validation Patient Set 1	Mutation	Male	33	2016	2.6	2	No	No	248.57	775.38	Beijing Tiantan Hospital
01P178	Validation Patient Set 1	Mutation	Male	45	911	2.5	1	No	No	261.43	364.40	Beijing Tiantan Hospital
01P110	Validation Patient Set 1	Mutation	Male	54	1366	2.9	2	No	No	362.71	471.03	Beijing Tiantan Hospital
01P084	Validation Patient Set 1	Mutation	Male	19	1078	3	1	No	No	388.71	359.33	Beijing Tiantan Hospital
01P063	Validation Patient Set 1	Mutation	Female	54	666	4.7	3	Yes	Yes	2.29	141.70	Beijing Tiantan Hospital
01P174	Validation Patient Set 1	Mutation	Male	40	1968	2.4	1	No	No	265.71	820.00	Beijing Tiantan Hospital
01P109	Validation Patient Set 1	Mutation	Male	60	236	2.5	1	No	No	363.29	94.40	Beijing Tiantan Hospital
01P237	Validation Patient Set 1	Mutation	Male	32	5550	3.7	3	Yes	No	164.57	1500.00	Beijing Tiantan Hospital
01P100	Validation Patient Set 1	Mutation	Male	38	6000	3.8	3	Yes	No	373.14	1578.95	Beijing Tiantan Hospital
01P137	Validation Patient Set 1	Mutation	Male	29	3961	2.6	1	No	No	326.71	1523.46	Beijing Tiantan Hospital
01P076	Validation Patient Set 1	Mutation	Male	26	754	3	1	No	Yes	373.00	251.33	Beijing Tiantan Hospital

01P082	Validation Patient Set 1	Wild	Male	50	2214	3.9	3	Yes	No	394.29	567.69	Beijing Tiantan Hospital
01P146	Validation Patient Set 1	Wild	Male	31	1776	2.4	4	Yes	N	N	740.00	Beijing Tiantan Hospital
01P171	Validation Patient Set 1	Wild	Female	51	1366	2.5	1	No	No	268.71	546.40	Beijing Tiantan Hospital
01P166	Validation Patient Set 1	Wild	Female	34	386	1.6	0	No	No	278.71	241.25	Beijing Tiantan Hospital
01P222	Validation Patient Set 1	Wild	Male	25	276	3	3	Yes	No	209.57	92.00	Beijing Tiantan Hospital
02P248	Validation Patient Set 1	Wild	Male	21	4392	4.1	4	Yes	No	99.00	1071.22	Sanbo Brain Hospital
02P153	Validation Patient Set 1	Wild	Male	48	489.8	1.7	2	No	No	302.43	288.12	Sanbo Brain Hospital
01P219	Validation Patient Set 1	Wild	Male	50	3226	3	3	Yes	No	214.71	1075.33	Beijing Tiantan Hospital
01P191	Validation Patient Set 1	Wild	Male	42	207	3.2	0	No	No	242.43	64.69	Beijing Tiantan Hospital
01P133	Validation Patient Set 1	Wild	Male	48	1604	3.5	3	Yes	No	335.71	458.29	Beijing Tiantan Hospital
01P143	Validation Patient Set 1	Wild	Male	33	474	2.8	3	Yes	No	309.71	169.29	Beijing Tiantan Hospital
01P132	Validation Patient Set 1	Wild	Male	48	2426	2.8	4	Yes	No	334.86	866.43	Beijing Tiantan Hospital
02P206	Validation Patient Set 1	Wild	Male	30	2118.6	3.3	2	No	No	223.00	642.00	Sanbo Brain Hospital
02P223	Validation Patient Set 1	Wild	Male	45	713	3.2	4	Yes	No	209.00	222.81	Sanbo Brain Hospital
01P142	Validation Patient Set 1	Wild	Male	25	6000	4.6	3	Yes	Yes	214.14	1304.35	Beijing Tiantan Hospital
01P077	Validation Patient Set 1	Wild	Male	29	271	2.5	2	No	No	407.43	108.40	Beijing Tiantan Hospital
02P187	Validation Patient Set 1	Wild	Female	21	160.7	1.1	1	No	No	248.29	146.09	Sanbo Brain Hospital
01P081	Validation Patient Set 1	Wild	Female	28	482	3.6	3	Yes	No	396.43	133.89	Beijing Tiantan Hospital
01P049	Validation Patient Set 1	Wild	Female	20	668	2.6	2	No	No	461.29	256.92	Beijing Tiantan Hospital
01P111	Validation Patient Set 1	Wild	Male	39	169	1.6	1	No	No	360.86	105.63	Beijing Tiantan Hospital
01P072	Validation Patient Set 1	Wild	Male	21	244	1.8	2	No	No	417.00	135.56	Beijing Tiantan Hospital

02P273	Validation Patient Set 1	Wild	Male	18	4000	2.5	3	Yes	No	28.00	1600.00	Sanbo Brain Hospital
01P085	Validation Patient Set 1	Wild	Male	46	531	2.7	3	Yes	No	388.29	196.67	Beijing Tiantan Hospital
01P096	Validation Patient Set 1	Wild	Male	45	349	2.3	2	No	No	377.71	151.74	Beijing Tiantan Hospital
02P236	Validation Patient Set 1	Wild	Female	18	290.5	3	2	No	No	167.00	96.83	Sanbo Brain Hospital
01P207	Validation Patient Set 1	Wild	Male	40	491	3.3	3	Yes	No	221.00	148.79	Beijing Tiantan Hospital
01P030	Validation Patient Set 1	Wild	Male	42	2741.2	3.5	3	Yes	N	N	783.20	Beijing Tiantan Hospital
02P246	Validation Patient Set 1	Wild	Male	37	115.04	1.9	2	No	No	109.14	60.55	Sanbo Brain Hospital
02P262	Validation Patient Set 1	Wild	Male	38	706.2	3.7	4	Yes	No	58.00	190.86	Sanbo Brain Hospital
02P240	Validation Patient Set 1	Wild	Female	17	2299	2.8	2	No	No	158.00	821.07	Sanbo Brain Hospital
01P105	Validation Patient Set 1	Wild	Female	51	217	2.7	2	No	No	372.86	80.37	Beijing Tiantan Hospital
02P261	Validation Patient Set 1	Wild	Male	32	611	3.1	4	Yes	No	70.29	197.10	Sanbo Brain Hospital
02P270	Validation Patient Set 1	Wild	Male	29	1134.8	4	4	Yes	No	35.14	283.70	Sanbo Brain Hospital
01P065	Validation Patient Set 1	Wild	Female	50	398	2.3	2	No	No	428.57	173.04	Beijing Tiantan Hospital
01P138	Validation Patient Set 1	Wild	Male	65	3196	2.4	3	Yes	N	N	1331.67	Beijing Tiantan Hospital
01P056	Validation Patient Set 1	Wild	Male	38	1277	4.2	4	Yes	Yes	30.86	304.05	Beijing Tiantan Hospital
01P067	Validation Patient Set 1	Wild	Male	29	6000	4.8	4	Yes	No	58.14	1250.00	Beijing Tiantan Hospital
01P058	Validation Patient Set 1	Wild	Male	33	214	1.5	0	No	No	435.57	142.67	Beijing Tiantan Hospital
01P062	Validation Patient Set 1	Wild	Male	49	919	2.2	1	No	No	432.71	417.73	Beijing Tiantan Hospital
01P112	Validation Patient Set 1	Wild	Male	55	2741	2.7	2	No	No	16.00	1015.19	Beijing Tiantan Hospital
01P073	Validation Patient Set 1	Wild	Female	30	778	1.4	2	No	No	415.29	555.71	Beijing Tiantan Hospital
01P239	Validation Patient Set 1	Wild	Male	27	1307	3.1	2	No	No	105.29	421.61	Beijing Tiantan Hospital

01P115	Validation Patient Set 1	Wild	Female	9	1192	3.6	2	No	No	354.86	331.11	Beijing Tiantan Hospital
01P120	Validation Patient Set 1	Wild	Female	40	532	2.4	2	No	Yes	10.71	221.67	Beijing Tiantan Hospital
01P052	Validation Patient Set 1	Wild	Female	23	364.2	2	3	Yes	N	N	182.10	Beijing Tiantan Hospital
01P103	Validation Patient Set 1	Wild	Male	32	2283	4.3	3	Yes	No	373.43	530.93	Beijing Tiantan Hospital
01P054	Validation Patient Set 1	Wild	Male	39	640	2	1	No	No	446.14	320.00	Beijing Tiantan Hospital
01P238	Validation Patient Set 1	Wild	Male	26	2255	4.4	4	Yes	No	162.71	512.50	Beijing Tiantan Hospital
01P198	Validation Patient Set 1	Wild	Male	23	486	2.6	1	No	No	234.14	186.92	Beijing Tiantan Hospital
01P033	Validation Patient Set 1	Wild	Female	24	272.2	1.5	1	No	N	N	181.47	Beijing Tiantan Hospital
01P113	Validation Patient Set 1	Wild	Male	40	1862	5.8	4	Yes	Yes	21.86	321.03	Beijing Tiantan Hospital
01P181	Validation Patient Set 1	Wild	Female	26	417	1.5	1	No	No	257.29	278.00	Beijing Tiantan Hospital
01P034	Validation Patient Set 1	Wild	Male	45	649.6	2.2	2	No	N	N	295.27	Beijing Tiantan Hospital
01P186	Validation Patient Set 1	Wild	Male	44	1062	3.6	3	Yes	No	250.57	295.00	Beijing Tiantan Hospital
01P148	Validation Patient Set 1	Wild	Male	11	429	5.4	2	No	No	305.57	79.44	Beijing Tiantan Hospital
01P169	Validation Patient Set 1	Wild	Male	43	385	2.5	3	Yes	No	267.57	154.00	Beijing Tiantan Hospital
01P114	Validation Patient Set 1	Wild	Female	23	291	1	1	No	No	355.86	291.00	Beijing Tiantan Hospital
01P149	Validation Patient Set 1	Wild	Male	32	4562	3.4	4	Yes	No	305.43	1341.76	Beijing Tiantan Hospital
01P095	Validation Patient Set 1	Wild	Female	29	1521	2.4	3	Yes	No	376.00	633.75	Beijing Tiantan Hospital
01P050	Validation Patient Set 1	Wild	Male	52	540	2.8	1	No	No	460.43	192.86	Beijing Tiantan Hospital
01P094	Validation Patient Set 1	Wild	Male	55	1490	2.8	4	Yes	N	N	532.14	Beijing Tiantan Hospital
01P108	Validation Patient Set 1	Wild	Male	52	1088	1.9	2	No	No	365.43	572.63	Beijing Tiantan Hospital
01P037	Validation Patient Set 1	Wild	Female	21	356.94	0.9	0	No	No	494.71	396.60	Beijing Tiantan Hospital

01P139	Validation Patient Set 1	Wild	Female	46	3432	4.2	4	Yes	No	235.43	817.14	Beijing Tiantan Hospital
01P225	Validation Patient Set 1	Wild	Male	12	6000	5.6	4	Yes	No	206.43	1071.43	Beijing Tiantan Hospital
01P098	Validation Patient Set 1	Wild	Female	37	215	1.5	1	No	No	374.29	143.33	Beijing Tiantan Hospital
01P212	Validation Patient Set 1	Wild	Male	39	2902	4.2	4	Yes	No	144.14	690.95	Beijing Tiantan Hospital
01P182	Validation Patient Set 1	Wild	Female	41	217	1.2	2	No	No	256.86	180.83	Beijing Tiantan Hospital
01P275	Validation Patient Set 1	Wild	Female	54	4148	4.5	2	No	No	17.14	921.78	Beijing Tiantan Hospital
01P131	Validation Patient Set 1	Wild	Male	53	6000	4.9	4	Yes	No	337.57	1224.49	Beijing Tiantan Hospital
01P044	Validation Patient Set 1	Wild	Female	31	1298	3.5	2	No	No	470.43	370.86	Beijing Tiantan Hospital
01P087	Validation Patient Set 1	Wild	Male	40	221	1.8	2	No	N	N	122.78	Beijing Tiantan Hospital
01P204	Validation Patient Set 1	Wild	Female	21	304	2.4	1	No	No	226.29	126.67	Beijing Tiantan Hospital
01P217	Validation Patient Set 1	Wild	Male	28	247	2.6	1	No	No	216.71	95.00	Beijing Tiantan Hospital
01P036	Validation Patient Set 1	Wild	Male	38	2296.8	3.7	4	Yes	N	N	620.76	Beijing Tiantan Hospital
01P216	Validation Patient Set 1	Wild	Male	15	223	1.9	0	No	No	216.57	117.37	Beijing Tiantan Hospital
01P210	Validation Patient Set 1	Wild	Male	43	1117	3.2	3	Yes	No	218.00	349.06	Beijing Tiantan Hospital
01P128	Validation Patient Set 1	Wild	Male	27	677	5.4	3	Yes	No	63.43	125.37	Beijing Tiantan Hospital
01P047	Validation Patient Set 1	Wild	Female	36	1061	2.8	3	Yes	No	464.43	378.93	Beijing Tiantan Hospital
01P185	Validation Patient Set 1	Wild	Female	13	2131	3.4	3	Yes	No	250.57	626.76	Beijing Tiantan Hospital
01P093	Validation Patient Set 1	Wild	Male	46	2522	4.1	2	No	No	379.71	615.12	Beijing Tiantan Hospital
01P147	Validation Patient Set 1	Wild	Male	50	745	3.2	4	Yes	N	N	232.81	Beijing Tiantan Hospital
01P117	Validation Patient Set 1	Wild	Male	68	1221	2.5	2	No	No	354.43	488.40	Beijing Tiantan Hospital
01P160	Validation Patient Set 1	Wild	Female	24	1016	1.8	1	No	No	289.00	564.44	Beijing Tiantan Hospital

01P228	Validation Patient Set 1	Wild	Female	37	2125	2	3	Yes	No	195.00	1062.50	Beijing Tiantan Hospital
01P127	Validation Patient Set 1	Wild	Female	21	405	1.9	2	No	No	193.29	213.16	Beijing Tiantan Hospital
01P211	Validation Patient Set 1	Wild	Male	42	1694	3.5	2	No	No	219.71	484.00	Beijing Tiantan Hospital
01P101	Validation Patient Set 1	Wild	Male	48	3811	4.2	3	Yes	No	372.43	907.38	Beijing Tiantan Hospital
01P176	Validation Patient Set 1	Wild	Male	38	2379	4.8	4	Yes	No	265.00	495.63	Beijing Tiantan Hospital
01P055	Validation Patient Set 1	Wild	Female	38	302	1.5	1	No	No	442.71	201.33	Beijing Tiantan Hospital
01P116	Validation Patient Set 1	Wild	Male	39	388	4	3	Yes	No	18.14	97.00	Beijing Tiantan Hospital
01P102	Validation Patient Set 1	Wild	Male	39	1074	3.2	4	Yes	No	374.43	335.63	Beijing Tiantan Hospital
01P064	Validation Patient Set 1	Wild	Female	34	208	1.6	0	No	No	428.71	130.00	Beijing Tiantan Hospital
01P135	Validation Patient Set 1	Wild	Female	28	679	2.4	2	No	No	332.43	282.92	Beijing Tiantan Hospital
01P168	Validation Patient Set 1	Wild	Male	54	896	2	1	No	No	270.57	448.00	Beijing Tiantan Hospital
01P177	Validation Patient Set 1	Wild	Male	15	579	1.7	2	No	No	264.00	340.59	Beijing Tiantan Hospital
01P097	Validation Patient Set 1	Wild	Female	24	600	2.8	2	No	N	N	214.29	Beijing Tiantan Hospital
01P079	Validation Patient Set 1	Wild	Female	57	4434	4.3	4	Yes	N	N	1031.16	Beijing Tiantan Hospital
01P048	Validation Patient Set 1	Wild	Female	31	455.4	1.3	2	No	N	N	350.31	Beijing Tiantan Hospital
01P123	Validation Patient Set 1	Wild	Male	45	533	2.5	1	No	No	344.43	213.20	Beijing Tiantan Hospital
01P152	Validation Patient Set 1	Wild	Male	18	358	2.6	3	Yes	No	303.00	137.69	Beijing Tiantan Hospital
01P136	Validation Patient Set 1	Wild	Male	65	295	3	3	Yes	No	330.29	98.33	Beijing Tiantan Hospital
01P200	Validation Patient Set 1	Wild	Female	42	386	1	0	No	No	230.00	386.00	Beijing Tiantan Hospital
01P196	Validation Patient Set 1	Wild	Male	54	1187	4.2	3	Yes	No	234.29	282.62	Beijing Tiantan Hospital
01P124	Validation Patient Set 1	Wild	Male	49	416	2.8	3	Yes	Yes	39.00	148.57	Beijing Tiantan Hospital

01P071	Validation Patient Set 1	Wild	Male	22	4000	4.6	3	Yes	Yes	118.00	869.57	Beijing Tiantan Hospital
01P165	Validation Patient Set 1	Wild	Male	30	2236	3.7	4	Yes	No	282.86	604.32	Beijing Tiantan Hospital
01P183	Validation Patient Set 1	Wild	Female	30	370	1.4	1	No	Yes	248.29	264.29	Beijing Tiantan Hospital
01P151	Validation Patient Set 1	Wild	Male	39	1175	3.7	3	Yes	No	304.00	317.57	Beijing Tiantan Hospital
01P039	Validation Patient Set 1	Wild	Female	31	344.2	1.5	0	No	No	482.57	229.47	Beijing Tiantan Hospital
01P134	Validation Patient Set 1	Wild	Female	59	260	3	3	Yes	No	335.14	86.67	Beijing Tiantan Hospital
01P230	Validation Patient Set 1	Wild	Male	22	548	3	4	Yes	No	191.29	182.67	Beijing Tiantan Hospital
01P029	Validation Patient Set 1	Wild	Male	53	1575.8	5.2	4	Yes	N	N	303.04	Beijing Tiantan Hospital
01P144	Validation Patient Set 1	Wild	Male	34	3533	4	4	Yes	N	N	883.25	Beijing Tiantan Hospital
01P066	Validation Patient Set 1	Wild	Female	24	431	3.2	1	No	No	17.86	134.69	Beijing Tiantan Hospital
01P173	Validation Patient Set 1	Wild	Male	31	3218	3.5	3	Yes	No	267.43	919.43	Beijing Tiantan Hospital
01P272	Validation Patient Set 1	Wild	Female	33	108	2.6	2	No	No	17.14	41.54	Beijing Tiantan Hospital
01P075	Validation Patient Set 1	Wild	Female	17	1526	3.2	2	No	No	15.14	476.88	Beijing Tiantan Hospital
01P130	Validation Patient Set 1	Wild	Male	31	752	4.6	4	Yes	Yes	27.14	163.48	Beijing Tiantan Hospital
01P220	Validation Patient Set 1	Wild	Male	42	3931	4.2	3	Yes	No	214.43	935.95	Beijing Tiantan Hospital
01P194	Validation Patient Set 1	Wild	Male	36	287	4.5	4	Yes	No	238.29	63.78	Beijing Tiantan Hospital
01P069	Validation Patient Set 1	Wild	Male	50	1079	2.3	1	No	No	418.57	469.13	Beijing Tiantan Hospital
01P199	Validation Patient Set 1	Wild	Female	25	312	1.3	1	No	No	13.86	240.00	Beijing Tiantan Hospital
01P164	Validation Patient Set 1	Wild	Male	32	767	2.2	2	No	No	19.00	348.64	Beijing Tiantan Hospital
01P180	Validation Patient Set 1	Wild	Male	33	1254	1.5	1	No	No	259.71	836.00	Beijing Tiantan Hospital
01P089	Validation Patient Set 1	Wild	Female	23	254	2	2	No	Yes	108.29	127.00	Beijing Tiantan Hospital

01P167	Validation Patient Set 1	Wild	Male	62	1183	3.4	3	Yes	No	269.57	347.94	Beijing Tiantan Hospital
01P202	Validation Patient Set 1	Wild	Male	35	2824	1.8	2	No	N	N	1568.89	Beijing Tiantan Hospital
01P080	Validation Patient Set 1	Wild	Male	65	631	3.2	3	Yes	No	398.57	197.19	Beijing Tiantan Hospital
01P140	Validation Patient Set 1	Wild	Male	57	326	2.5	2	No	No	314.86	130.40	Beijing Tiantan Hospital
01P154	Validation Patient Set 1	Wild	Male	11	759	3.3	2	No	No	301.43	230.00	Beijing Tiantan Hospital
01P192	Validation Patient Set 1	Wild	Female	18	300	1.6	2	No	No	242.29	187.50	Beijing Tiantan Hospital
01P078	Validation Patient Set 1	Wild	Female	47	293	1.5	2	No	No	408.29	195.33	Beijing Tiantan Hospital
01P032	Validation Patient Set 1	Wild	Female	21	427	1.4	0	No	No	516.86	305.00	Beijing Tiantan Hospital
01P159	Validation Patient Set 1	Wild	Female	54	1222	2.3	3	Yes	No	290.29	531.30	Beijing Tiantan Hospital
02P205	Validation Patient Set 1	Wild	Female	32	308	1.2	2	No	No	224.86	256.67	Sanbo Brain Hospital
02P203	Validation Patient Set 1	Wild	Female	31	97.32	0.5	2	No	No	227.86	194.64	Sanbo Brain Hospital
02P172	Validation Patient Set 1	Wild	Male	17	3514.6	2	4	Yes	No	267.00	1757.30	Sanbo Brain Hospital
02P247	Validation Patient Set 1	Wild	Male	52	104	4	4	Yes	No	100.43	26.00	Sanbo Brain Hospital
02P227	Validation Patient Set 1	Wild	Female	34	136.64	0.8	0	No	No	203.29	170.80	Sanbo Brain Hospital
03P004	Validation Patient Set 2	Mutation	Male	55	> 200				N	N		The First Affiliated Hospital of USTC
03P001	Validation Patient Set 2	Mutation	Male	67	>200.00	3			N	N		The First Affiliated Hospital of USTC
03P002	Validation Patient Set 2	Mutation	Male	45	> 205	5			N	N		The First Affiliated Hospital of USTC
03P003	Validation Patient Set 2	Mutation	Male	65	525.3	3.6			N	N		The First Affiliated Hospital of USTC
03P005	Validation Patient Set 2	Mutation	Male	38	>200.00				N	N		The First Affiliated Hospital of USTC
03P011	Validation Patient Set 2	Wild	Female	21	122.97	2.3			N	N		The First Affiliated Hospital of USTC
03P007	Validation Patient Set 2	Wild	Male	29	>200.00	3.5			N	N		The First Affiliated Hospital of USTC

03P006	Validation Patient Set 2	Wild	Male	33	>209.00	4		N	N	The First Affiliated Hospital of USTC
03P008	Validation Patient Set 2	Wild	Male	69	1263	3.4		N	N	The First Affiliated Hospital of USTC
03P016	Validation Patient Set 2	Wild	Female	16	193.87	2		N	N	The First Affiliated Hospital of USTC
03P023	Validation Patient Set 2	Wild	Male	60	>200.00	3		N	N	The First Affiliated Hospital of USTC
03P027	Validation Patient Set 2	Wild	Male	62	＞209			N	N	The First Affiliated Hospital of USTC
03P017	Validation Patient Set 2	Wild	Male	22	>200.00	2		N	N	The First Affiliated Hospital of USTC
03P019	Validation Patient Set 2	Wild	Female	24	>200.00			N	N	The First Affiliated Hospital of USTC
03P025	Validation Patient Set 2	Wild	Male	70	>200.00	2.5		N	N	The First Affiliated Hospital of USTC
03P024	Validation Patient Set 2	Wild	Male	49	686.4	2.7		N	N	The First Affiliated Hospital of USTC
03P015	Validation Patient Set 2	Wild	Male	41	192.98	2		N	N	The First Affiliated Hospital of USTC
03P012	Validation Patient Set 2	Wild	Female	56	>209.00	2.3		N	N	The First Affiliated Hospital of USTC
03P013	Validation Patient Set 2	Wild	Male	58	>209.00	2.2		N	N	The First Affiliated Hospital of USTC
03P028	Validation Patient Set 2	Wild	Male	43	＞209			N	N	The First Affiliated Hospital of USTC
03P018	Validation Patient Set 2	Wild	Female	60	>200.00	1.7		N	N	The First Affiliated Hospital of USTC
03P010	Validation Patient Set 2	Wild	Male	66	2717	2.6		N	N	The First Affiliated Hospital of USTC
03P021	Validation Patient Set 2	Wild	Female	63	>209.00	3.2		N	N	The First Affiliated Hospital of USTC
03P009	Validation Patient Set 2	Wild	Male	38	3083	3.2		N	N	The First Affiliated Hospital of USTC
03P026	Validation Patient Set 2	Wild	Female	44	>200.00	2.4		N	N	The First Affiliated Hospital of USTC
03P022	Validation Patient Set 2	Wild	Male	66	>200.00	3.2		N	N	The First Affiliated Hospital of USTC
03P014	Validation Patient Set 2	Wild	Female	46	711	2.2		N	N	The First Affiliated Hospital of USTC
03P020	Validation Patient Set 2	Wild	Male	69	>200.00			N	N	The First Affiliated Hospital of USTC

*Tumor progression was defined when a patient developed postoperative symptomatic, biochemical or radiological recurrence.

#PFS: Progression Free Survival.

In validation patient set 2, N means no available data.

USTC, University of Science and Technology of China; FFPE, formalin-fixed paraffin embedded; DDPCR, Droplet digital PCR

Supplementary Table 8 Primers used for CLIP-PCR.

Gene ID	Transcript ID	Location	Primer ID	Sequence (5'-3')
2104	NM_001243518.1	6-25	P1	Forward: GCGAGAATGTGATTGGGGTC
				Reverse: GCTGTCAACGATACGCTACG
2104	NM_001243518.1	112-131	P2	Forward: TCCAGCTGTTCGTCCTTCAT
				Reverse: GCTGTCAACGATACGCTACG
2104	NM_001243518.1	231-250	P3	Forward: GAATGGCCATCAGAACGGAC
				Reverse: GCTGTCAACGATACGCTACG
2104	NM_001243518.1	345-364	P4	Forward: AGATCCCCAGACCAAGTGTG
				Reverse: GCTGTCAACGATACGCTACG
2104	NM_001243518.1	409-428	P5	Forward: GGTGACATCGCTTCTGGGTA
				Reverse: GCTGTCAACGATACGCTACG
2104	NM_001243518.1	506-524	P6	Forward: GCCCTGCCACGAATGAATG
				Reverse: GCTGTCAACGATACGCTACG
2104	NM_001243518.1	628-647	P7	Forward: GGAGGTCGGCAGAAGTACAA
				Reverse: GCTGTCAACGATACGCTACG
2104	NM_001243518.1	694-713	P8	Forward: TTGCTCTGGTCTGATCCTGC
				Reverse: GCTGTCAACGATACGCTACG
2104	NM_001243518.1	715-734	P9	Forward: GTTCAGCCAGCCAAAAAGCC
				Reverse: GCTGTCAACGATACGCTACG
2104	NM_001243518.1	738-762	P10	Forward:TAACAAGATTGTCTCACATTTGTTG
				Reverse: GCTGTCAACGATACGCTACG
2104	NM_001243518.1	765-784	P11	Forward: GGCTGAACCGGAGAAGATCT
				Reverse: GCTGTCAACGATACGCTACG
2104	NM_001243518.1	901-918	P12	Forward: TTCTCCACGCTGTCCCTG
				Reverse: GCTGTCAACGATACGCTACG
2104	NM_001243518.1	981-1000	P13	Forward: CCGGTCTCTTTCGTTTGAGG
				Reverse: GCTGTCAACGATACGCTACG
2104	NM_001243518.1	1082-1101	P14	Forward: TCCTGCAGCTGGTAAAGAAA
				Reverse: GCTGTCAACGATACGCTACG
2104	NM_001243518.1	1233-1252	P15	Forward: GCAGGATTATGAAGCTGGCC
				Reverse: GCTGTCAACGATACGCTACG
2104	NM_001243518.1	1291-1310	P16	Forward: ATGACACTGCCACTCCTGAG
				Reverse: GCTGTCAACGATACGCTACG
2104	NM_001243518.1	1327-1346	P17	Forward: GCCGTGCAGCATTTCTACAA
				Reverse: GCTGTCAACGATACGCTACG

Gene ID	Transcript ID	Location	Primer ID	Primer
2104	NM_001243519.1	28-47	P2	Forward: TCCAGCTGTTCGTCCTTCAT
				Reverse: GCTGTCAACGATACGCTACG
2104	NM_001243519.1	147-166	P3	Forward: GAATGGCCATCAGAACGGAC
				Reverse: GCTGTCAACGATACGCTACG
2104	NM_001243519.1	261-280	P4	Forward: AGATCCCCAGACCAAGTGTG
				Reverse: GCTGTCAACGATACGCTACG
2104	NM_001243519.1	325-344	P5	Forward: GGTGACATCGCTTCTGGGTA
				Reverse: GCTGTCAACGATACGCTACG
2104	NM_001243519.1	422-440	P6	Forward: GCCCTGCCACGAATGAATG
				Reverse: GCTGTCAACGATACGCTACG
2104	NM_001243519.1	544-563	P7	Forward: GGAGGTCGGCAGAAGTACAA
				Reverse: GCTGTCAACGATACGCTACG
2104	NM_001243519.1	610-629	P9	Forward: GTTCAGCCAGCCAAAAAGCC
				Reverse: GCTGTCAACGATACGCTACG
2104	NM_001243519.1	633-657	P10	Forward:TAACAAGATTGTCTCACATTTGTTG
				Reverse: GCTGTCAACGATACGCTACG
2104	NM_001243519.1	660-679	P11	Forward: GGCTGAACCGGAGAAGATCT
				Reverse: GCTGTCAACGATACGCTACG
2104	NM_001243519.1	796-813	P12	Forward: TTCTCCACGCTGTCCCTG
				Reverse: GCTGTCAACGATACGCTACG
2104	NM_001243519.1	876-895	P13	Forward: CCGGTCTCTTTCGTTTGAGG
				Reverse: GCTGTCAACGATACGCTACG
2104	NM_001243519.1	977-996	P14	Forward: TCCTGCAGCTGGTAAAGAAA
				Reverse: GCTGTCAACGATACGCTACG
2104	NM_001243519.1	1128-1147	P15	Forward: GCAGGATTATGAAGCTGGCC
				Reverse: GCTGTCAACGATACGCTACG
2104	NM_001243519.1	1186-1205	P16	Forward: ATGACACTGCCACTCCTGAG
				Reverse: GCTGTCAACGATACGCTACG
2104	NM_001243519.1	1222-1241	P17	Forward: GCCGTGCAGCATTTCTACAA
				Reverse: GCTGTCAACGATACGCTACG
2104	NM_001243519.1	619-643	P18	Forward:GCCAAAAAGCCATATAACAAGATTG
				Reverse: GCTGTCAACGATACGCTACG

Supplementary Table 9 Primers for qRT-PCR and siRNA.

a, Primers used for qRT-PCR analyses

Gene	Primer
SF3B1-1- forward	5′- GAAGGACGAGGCTGAGCAAT -3′
SF3B1-1-reverse	5′- GCGTACAGGATTCCGTCGAT -3′
SF3B1-2- forward	5′- TCCAAAGATTGCAGACCGGG-3′
SF3B1-2-reverse	5′-GATCAGGGGTTTTCCCTCCA-3′

Gene	Transcript ID	Location	Sequence (5'-3')
ESRRG (canonical)	NM_001243519.1	541-562	Forward: CGTGGAGGTCGGCAGAAGTACA
		759-779	Reverse: GCCCATCCAATGATAACCACC
ESRRG (cryptic)	NM_001243518.1	615-636	Forward: TGACAGAGTACGTGGAGGTCGG
		714-736	Reverse: CTGCAGGATCAGACCAGAGCAAT

b, Sequence of siRNA

Gene	Sequence
SF3B1-1-sense	5'- GCACAGACCUCCAAAGAUUTT -3'
SF3B1-1-antisense	5'- AAUCUUUGGAGGUCUGUGCTT -3'
SF3B1-2-sense	5'- CCUGAUGAUAUUCAAUACUTT-3'
SF3B1-2-antisense	5'- AGUAUUGAAUAUCAUCAGGTT-3'
ESRRG-1-sense	5'-GCCGCUUCAUGAAGUGUUUTT-3'
ESRRG-1-antisense	5'-AAACACUUCAUGAAGCGGCTT-3'
ESRRG-2--sense	5'- GCAAAGUCCCAAUGCACAATT-3'
ESRRG-2-antisense	5'- UUGUGCAUUGGGACUUUGCTT-3'
Negative control-sense	5'-UUCUCCGAACGUGUCACGUTT-3'
Negative control-antisense	5'-ACGUGACACGUUCGGAGAATT-3'

Supplementary Table 10 PCR conditions for the validation of cryptic splicing events in selected genes identified by rMATS.

Gene	Primer Sequence	Product Size	PCR Conditions
THRA (A3SS)	5'-CAACCACCGCAAACACAACA-3'<br>5'- CGGCCCTTGGAGACTTC-3'	Normal splicing 229bp;<br>aberrant splicing 112bp	Initial denaturation 98℃ for 2 min<br>98℃ 10 sec,<br>59℃ 10 sec,<br>72℃ 10 sec<br>for 32 cycles<br>final extension of 72℃ for 5 min
ANKHD1 (A3SS)	5'-CAGAAGGAGCAGACTTACGCACTG -3'<br>5'- CTGTTTTCTGCTTTCATCCGTGTC -3'	Normal splicing 205bp;<br>aberrant splicing 172bp	
MED6 (A3SS)	5'- TCATGATCTTTGAAGTGCCACCAAT -3'<br>5'- TGCTGGAGTGATCTATCAGGCACC -3'	Normal splicing 156bp;<br>aberrant splicing 177bp	
TMEM14C (A3SS)	5'-CAACCACCGCAAACACAACA-3'<br>5'- CGGCCCTTGGAGACTTC-3'	Normal splicing 181bp;<br>aberrant splicing 195bp	
MTERF2 (A3SS)	5'-CTCTCCCCACAGATCTTGTCCT-3'<br>5'- ACTGTCGGGAGCTTCTTGAGAC-3'	Normal splicing 253bp;<br>aberrant splicing 243bp	
ESRRG (A3SS)	5'-GGAATATGCTTCGCCCATCCAA-3'<br>5'- AGAGTACGTGGAGGTCGGCAGA-3'	Normal splicing 257bp;<br>aberrant splicing 278bp	
DYNLL1 (A3SS)	5'-CTTAGATGCGCCACGGTTTC-3'<br>5'- ATGAGCCGCAATGTCCTTCT-3'	Normal splicing 236bp;<br>aberrant splicing 250bp	
NCOA7 (A3SS)	5'-GGTTGTTTCCATTGCGCTCTAC-3'<br>5'- ATTGGTCTGGAATCCGAAGTCA-3'	Normal splicing 222bp;<br>aberrant splicing 189bp	
APBB3 (A3SS)	5'-TGGCACTTGAGCATACAGC-3'<br>5'- GATGCCATGAGCCTAGTGAA-3'	Normal splicing 176bp;<br>aberrant splicing 170bp	
FAM208A (A3SS)	5'-TTTTTGATCTGTTCCACGTGATCCTT-3'<br>5'-TGGGCTATCACAATTCAATCACAGAA-3'	Normal splicing 495bp;<br>aberrant splicing 218bp	
ZBED5 (A3SS)	5'- TCAATGCGCAGATGGAACATCA -3'<br>5'- AAGCTGCCAAGGTGGAGAGAGG -3'	Normal splicing 139bp;<br>aberrant splicing 171bp	
ORAI2 (A3SS)	5'-CCGGCGGAAGGAGGGAGGGG-3'<br>5'- CCGTGGTGCAGGCGCTGAAGGCAA-3'	Normal splicing 545bp;<br>aberrant splicing 568bp	
TMEM14C (MXE)	5'-CACTCTCCACCCGCTGAATG-3'<br>5'-ACCCAGGCCGGCTAGACTGC-3'	Normal splicing 439bp;<br>aberrant splicing 453bp	
DCAF16 (SE)	5'-ATGCCCTTGGAAACTGGTGA-3'<br>5'-AGGGCTCCGCTTCGAGAGAC-3'	Normal splicing 186bp;<br>aberrant splicing 222bp	
ABCC5 (SE)	5'-ACACCTCGGCACTGATGGAG-3'<br>5'- AATGAAGTTGGGCCAGACGC-3'	Normal splicing 158bp;<br>aberrant splicing 232bp	

Supplementary Table 11 Sequence of TaqMan MGB probes and primers in ddPCR.

Gene	Sequence
SF3B1-PA	HEX-5'-ATGTCCATAACACAAC -3'-MGB
SF3B1-PG	FAM-5'-AGTATGTCCGTAACACA-3'-MGB
SF3B1-PF	5'- CTGGCTACTATGATCTCTACCATGAGA-3'
SF3B1-PR	5'- GAGGCTACAACAGCAAAAGCTCTA-3'

Supplementary Table 12 The different vector transfection groups in luciferase reporter assays.

No.	Transfection vectors combination
**1**	pCDH+pCDNA3.1+pGL3-basic+pRL-TK
**2**	pCDH+pCDNA3.1-Pit-1+pGL3-basic+pRL-TK
**3**	pCDH-ESRRG-cryptic+pCDNA3.1+pGL3-basic+pRL-TK
**4**	pCDH-ESRRG-cryptic+pCDNA3.1-Pit-1+pGL3-basic+pRL-TK
**5**	pCDH-ESRRG-cryptic+pCDNA3.1+pGL3-basic-PRL-p+pRL-TK
**6**	pCDH-ESRRG-canonical+pCDNA3.1+pGL3-basic+pRL-TK
**7**	pCDH-ESRRG-canonical+pCDNA3.1-Pit-1+pGL3-basic+pRL-TK
**8**	pCDH-ESRRG-canonical+pCDNA3.1+pGL3-basic-PRL-p+pRL-TK
**9**	pCDH+pCDNA3.1+pGL3-basic-pRL-p+pRL-TK
**10**	pCDH+pCDNA3.1-Pit-1+pGL3-basic-pRL-p+pRL-TK
**11**	pCDH-ESRRG-canonical+pCDNA3.1-Pit-1+pGL3-basic-PRL-p+pRL-TK
**12**	pCDH-ESRRG-cryptic+pCDNA3.1-Pit-1+pGL3-basic-PRL-p+pRL-TK

References

1. Li H, Durbin R. Fast and accurate short read alignment with Burrows-Wheeler transform. Bioinformatics 2009;25:1754-60.

2. Li H, Handsaker B, Wysoker A, et al. The Sequence Alignment/Map format and SAMtools. Bioinformatics 2009;25:2078-9.

3. DePristo MA, Banks E, Poplin R, et al. A framework for variation discovery and genotyping using next-generation DNA sequencing data. Nat Genet 2011;43:491-8.

4. Cibulskis K, Lawrence MS, Carter SL, et al. Sensitive detection of somatic point mutations in impure and heterogeneous cancer samples. Nat Biotechnol 2013;31:213-9.

5. Saunders CT, Wong WS, Swamy S, Becq J, Murray LJ, Cheetham RK. Strelka: accurate somatic small-variant calling from sequenced tumor-normal sample pairs. Bioinformatics 2012;28:1811-7.

6. Trapnell C, Pachter L, Salzberg SL. TopHat: discovering splice junctions with RNA-Seq. Bioinformatics 2009;25:1105-11.

7. Trapnell C, Williams BA, Pertea G, et al. Transcript assembly and quantification by RNA-Seq reveals unannotated transcripts and isoform switching during cell differentiation. Nat Biotechnol 2010;28:511-5.
