# Supplementary material for: Somatic SF3B1 hotspot mutation in prolactinomas
Source: Nat Commun. 2020 May 19;11:2506. doi: 10.1038/s41467-020-16052-8 (PMC7237453; doi:10.1038/s41467-020-16052-8)

Fig. 2b

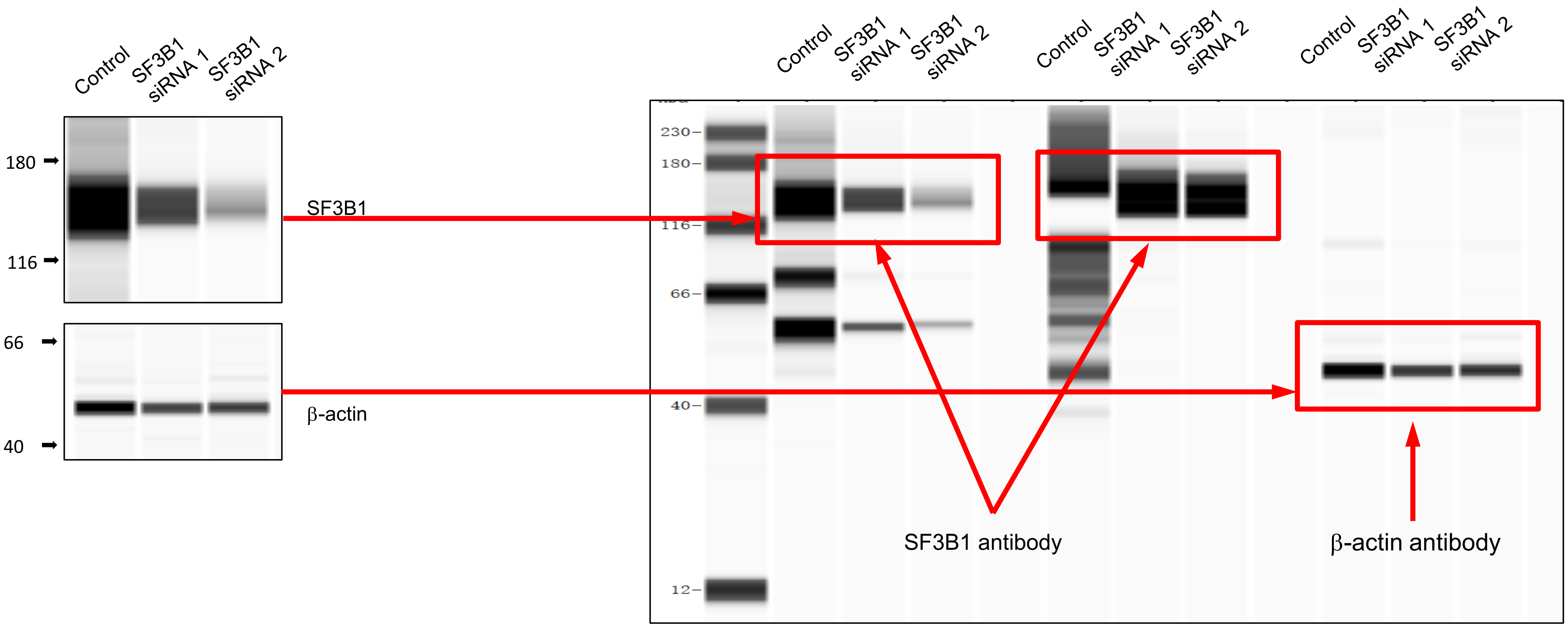
Control
SF3B1 siRNA 1
SF3B1 siRNA 2
180
116
66
40
SF3B1
β-actin
230
180
116
66
40
12
SF3B1 antibody
β-actin antibody

Fig.3c

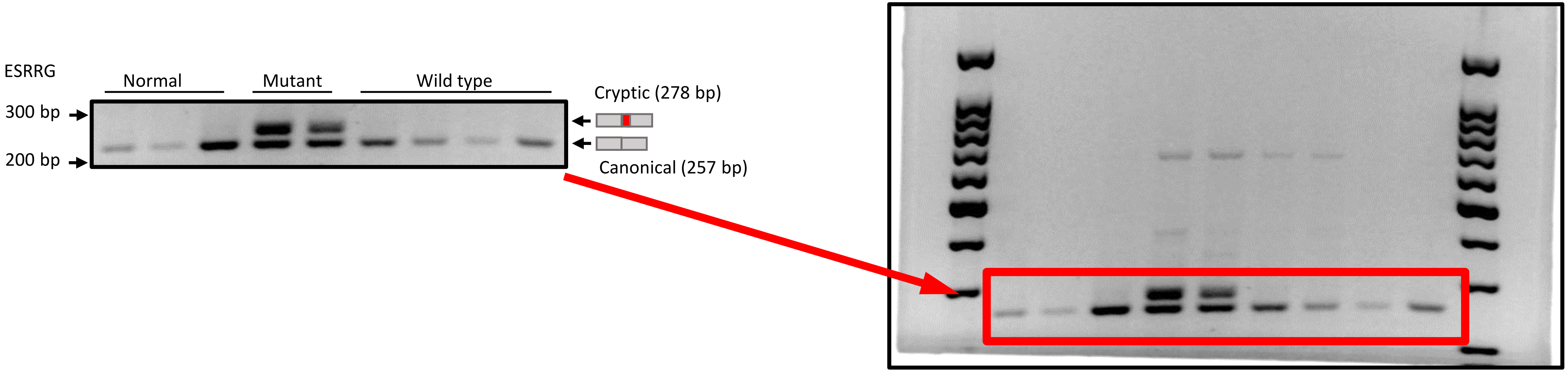
ESRRG
Normal
Mutant
Wild type
300 bp
200 bp
Cryptic (278 bp)
Canonical (257 bp)

Fig.3f

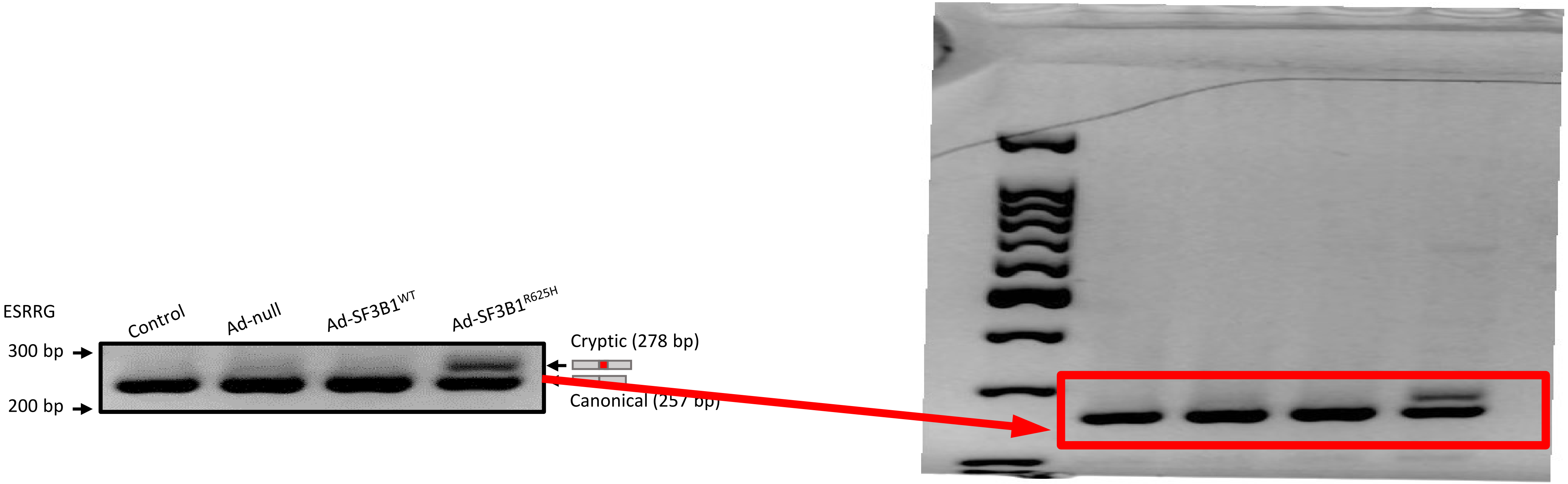

ESRRG
Control
Ad-null
Ad-SF3B1WT
Ad-SF3B1R625H
300 bp
200 bp
Cryptic (278 bp)
Canonical (257 bp)

Fig.4a

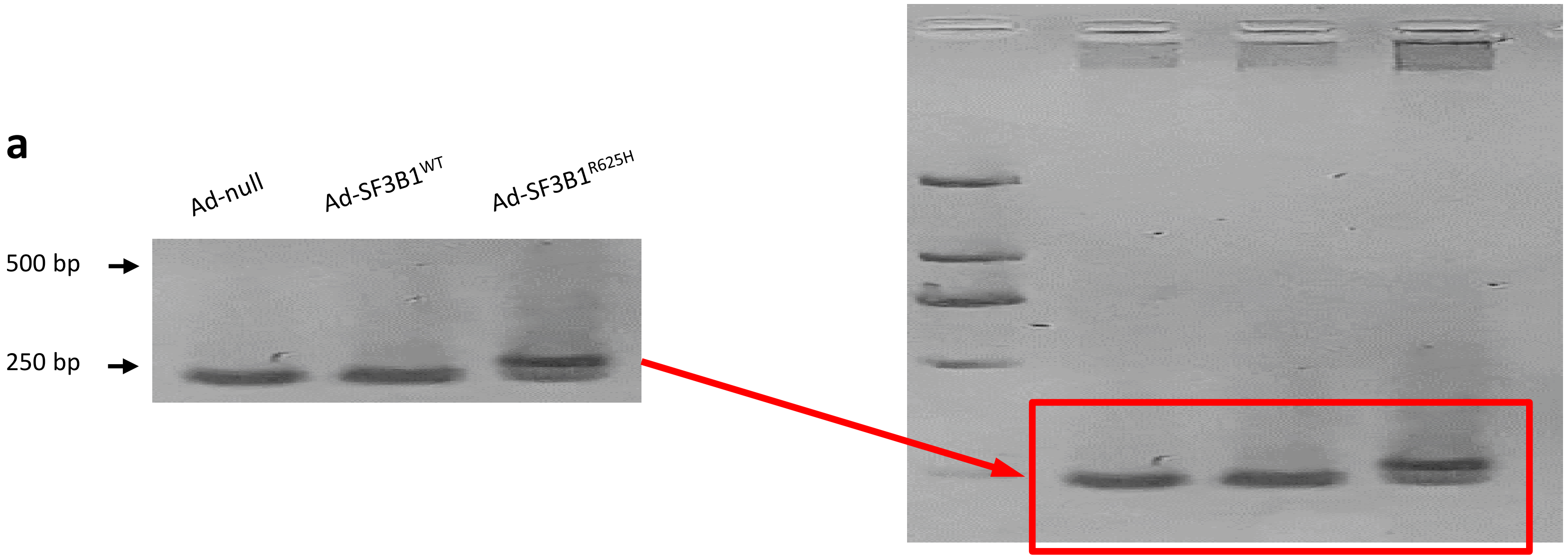

Fig.4e

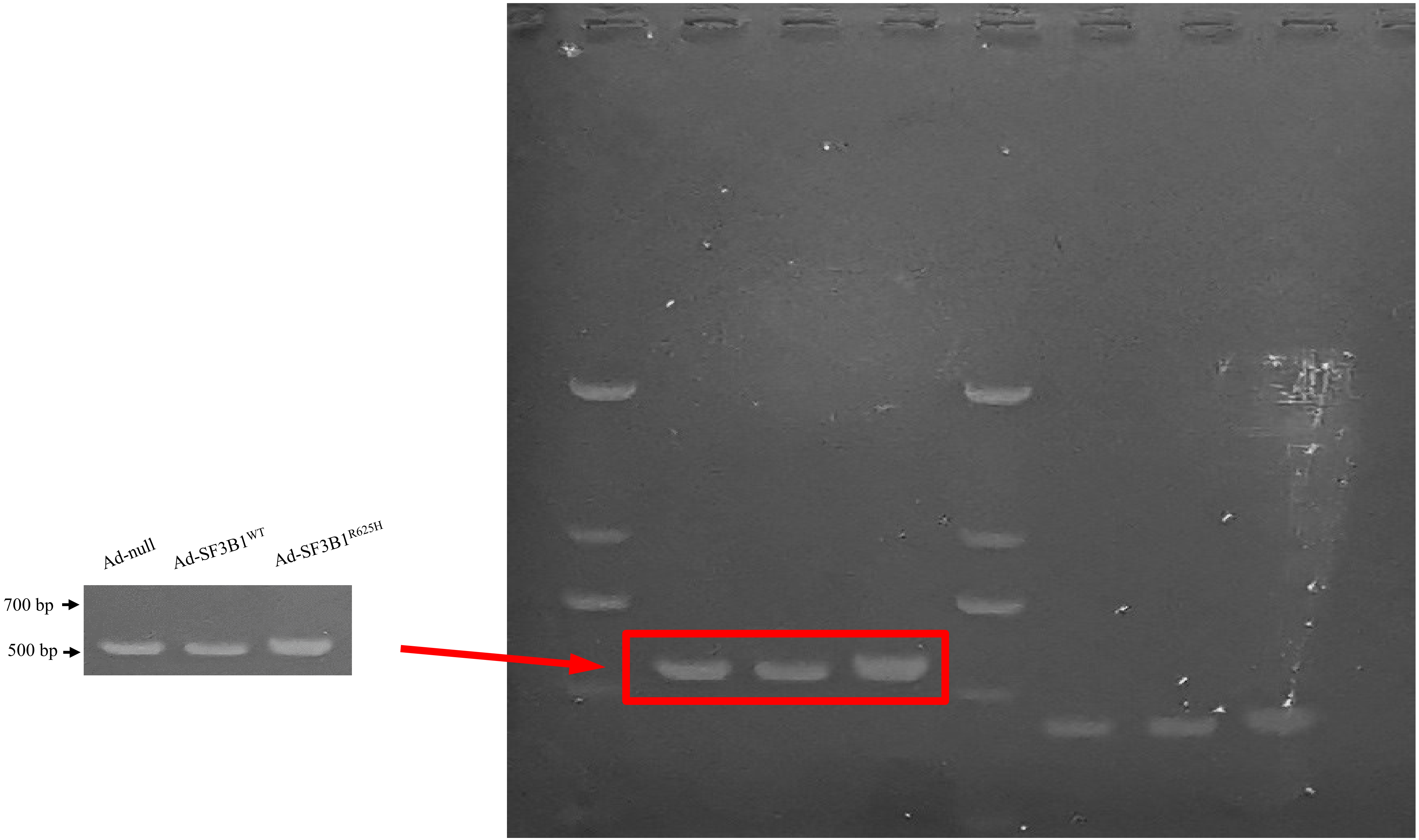

Fig.5f

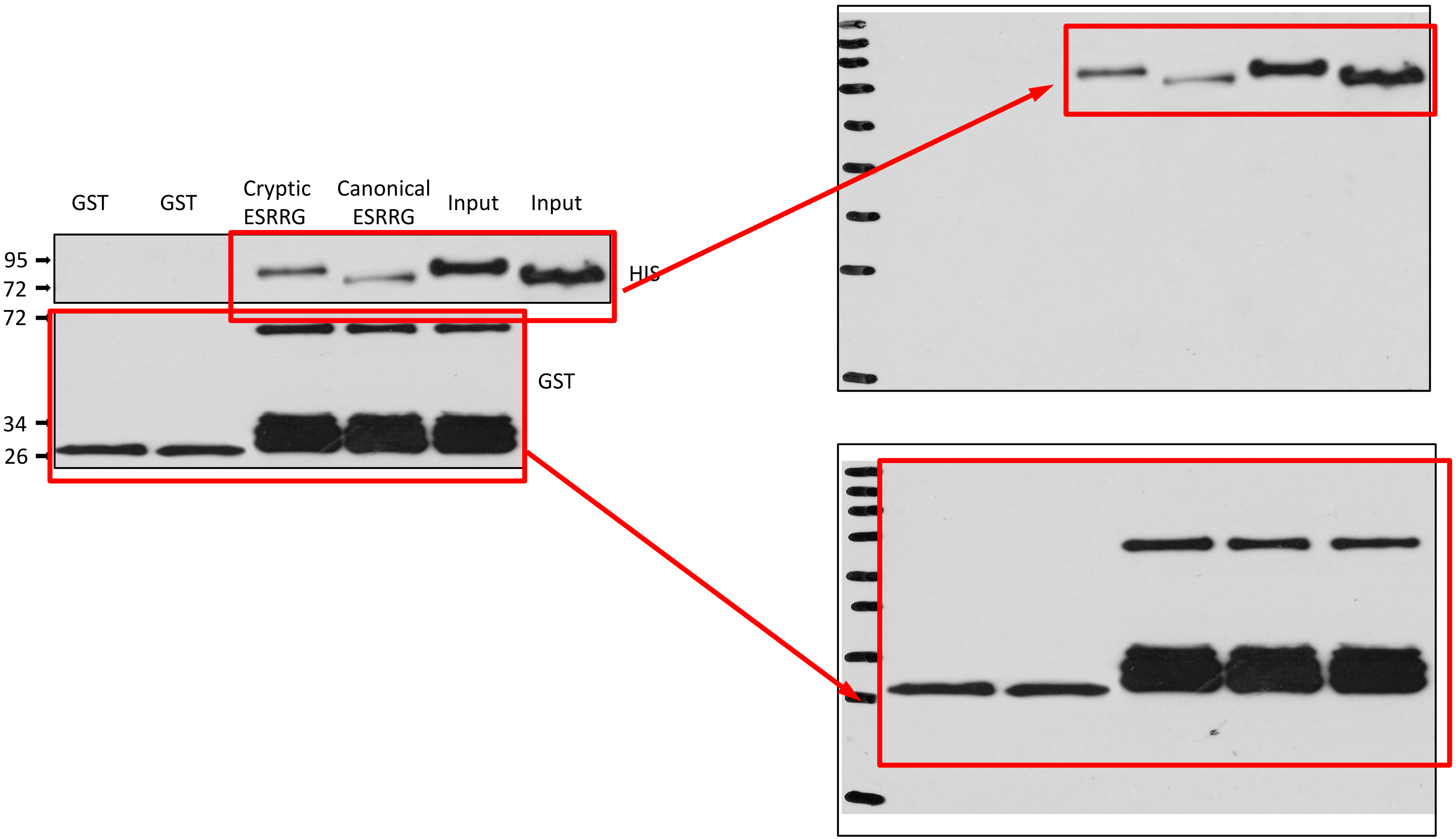
GST
GST
Cryptic ESRRG
Canonical ESRRG
Input
Input
95
72
72
34
26
HIS
GST

Fig.5g

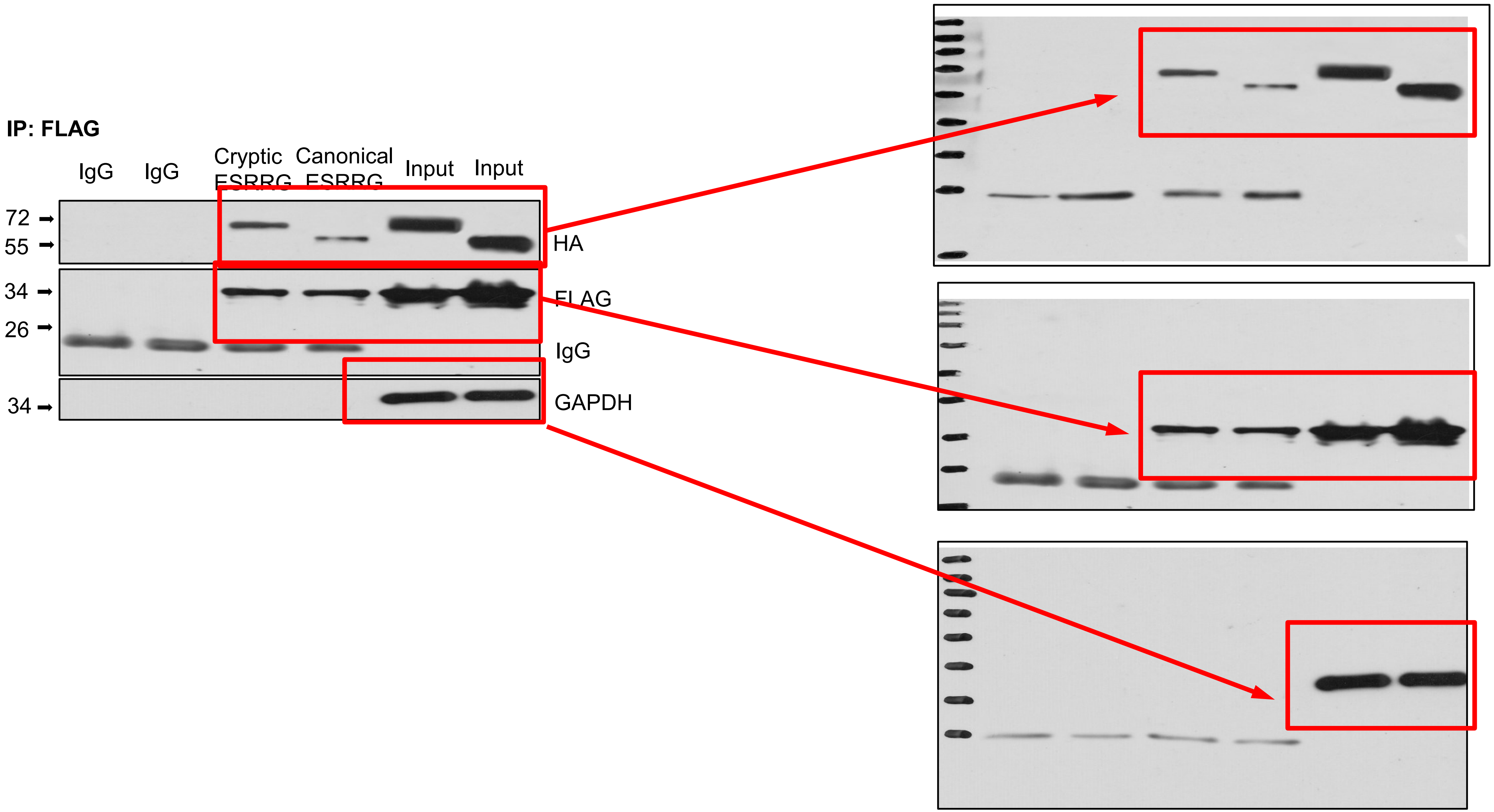
IP: FLAG
IgG
IgG
Cryptic ESRRG
Canonical ESRRG
Input
Input
72
55
34
26
34
HA
FLAG
IgG
GAPDH

Supplementary Fig. 4a

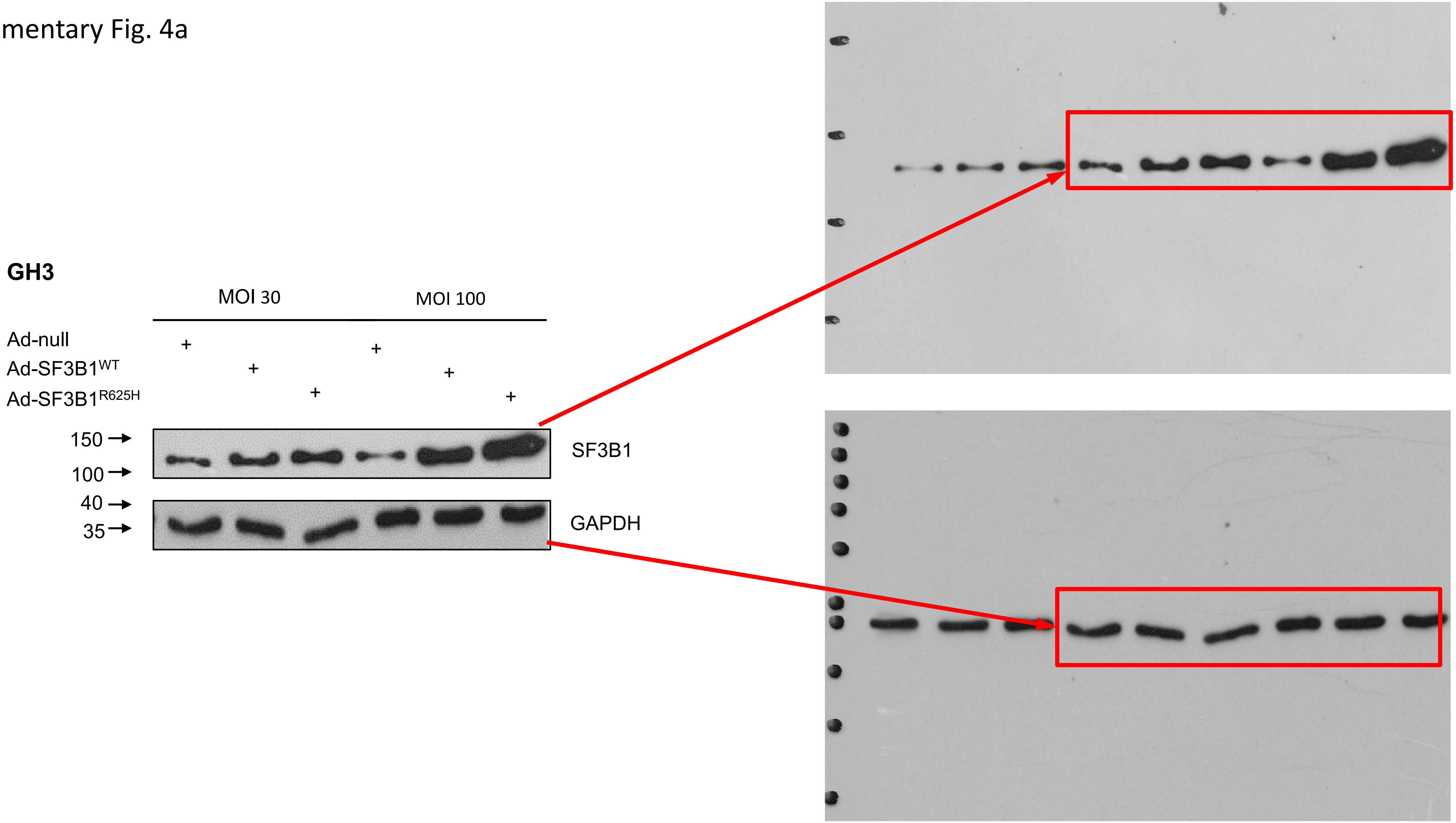

Supplementary Fig. 4b

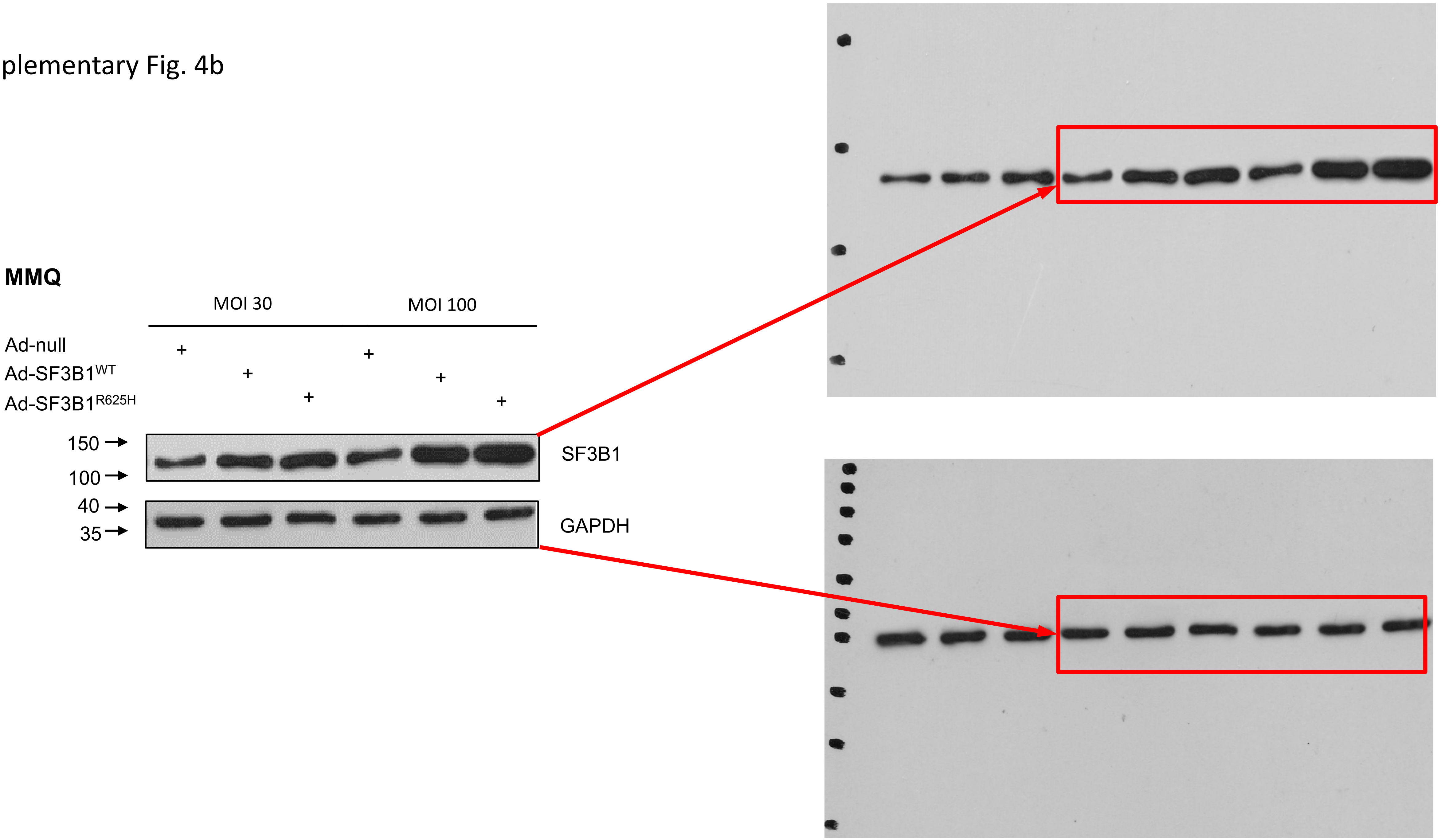

Supplementary Fig. 7

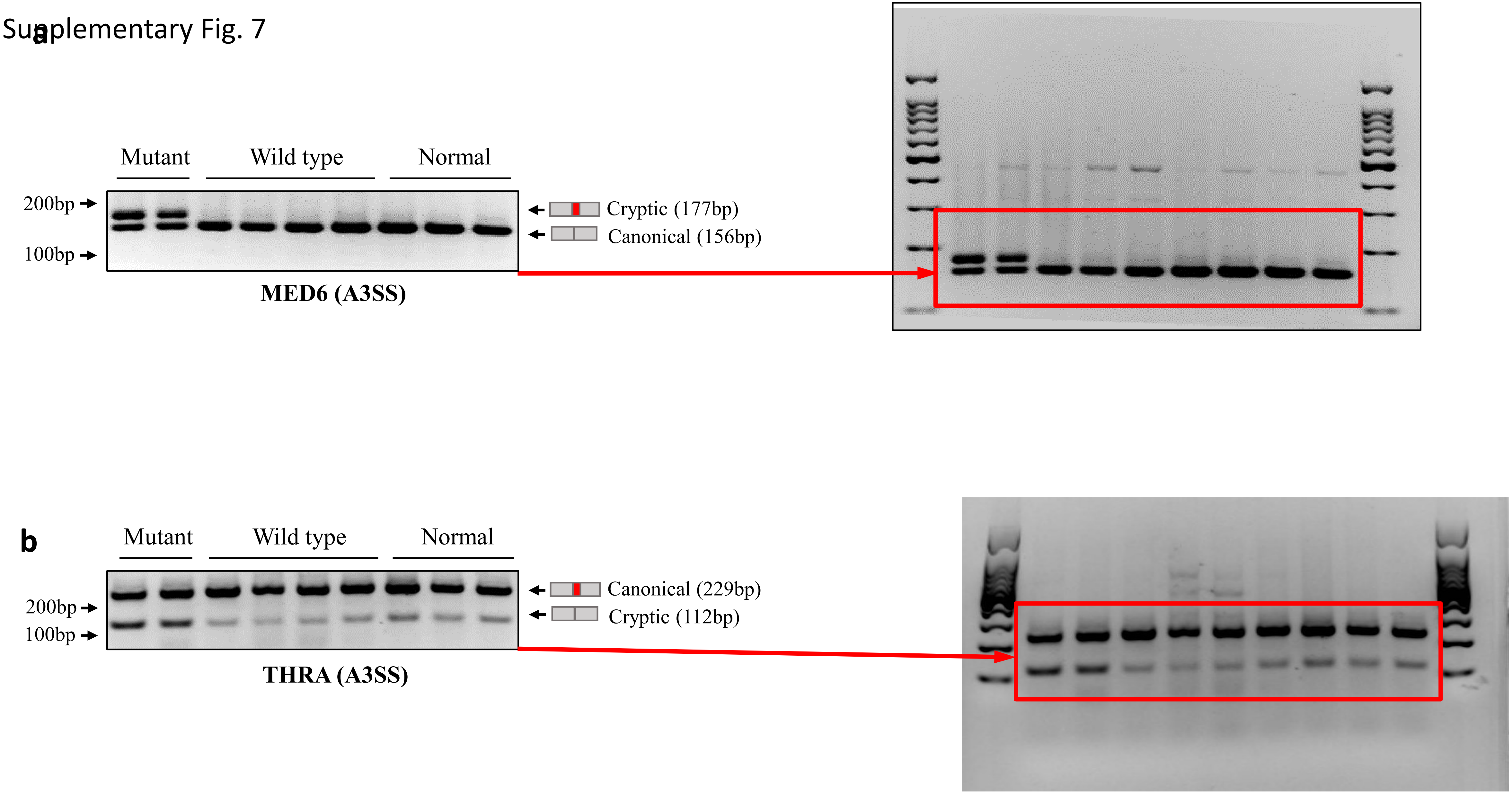

Supplementary Fig. 7

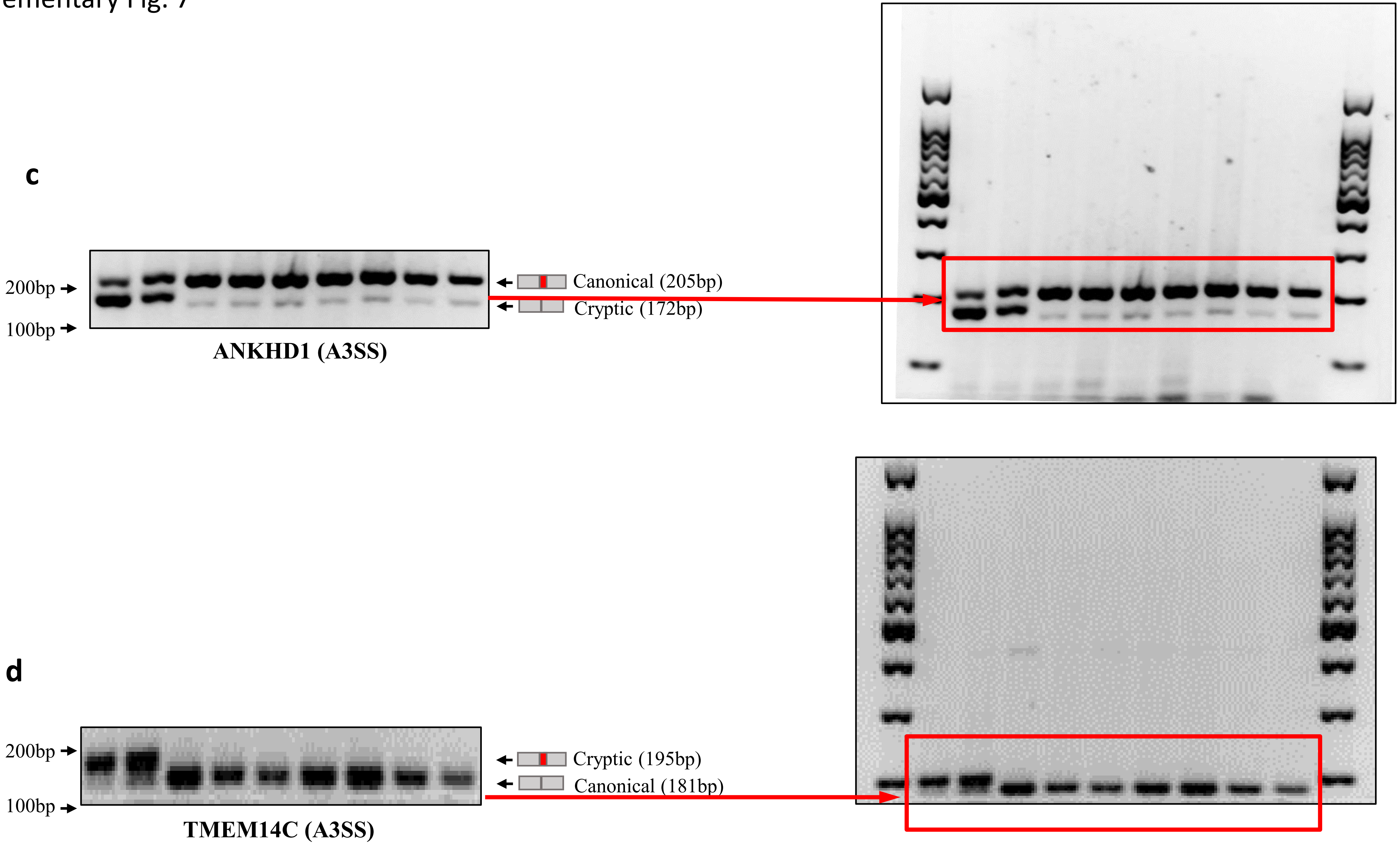

Supplementary Fig. 7

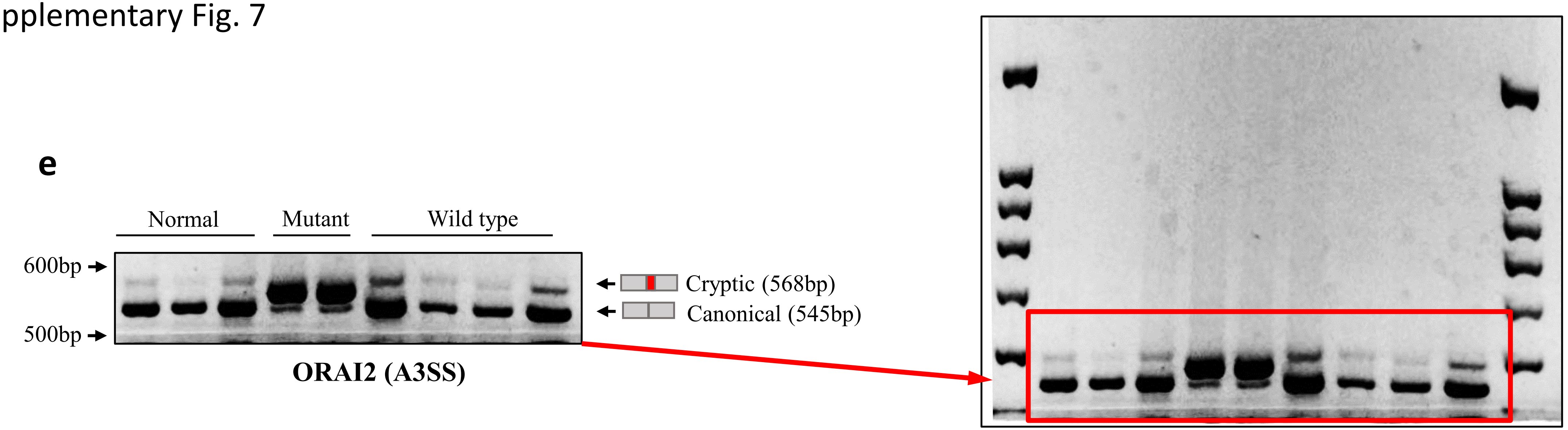

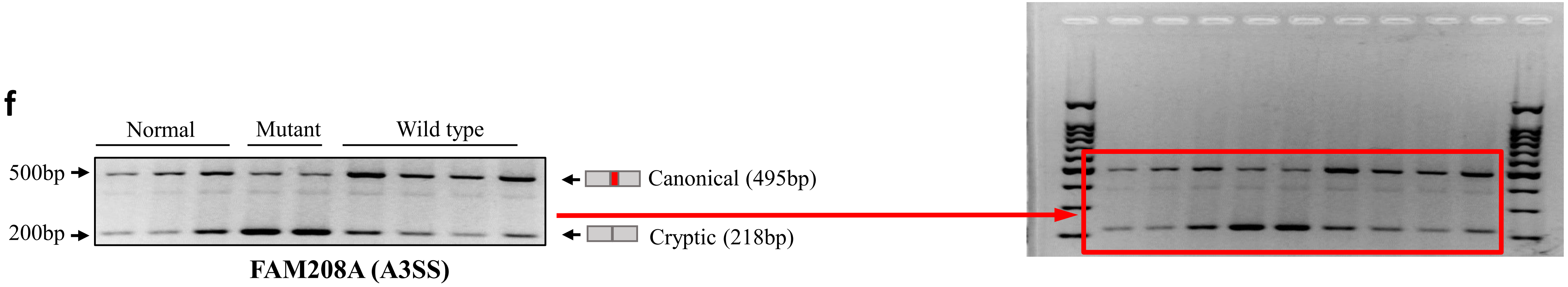

Supplementary Fig. 7

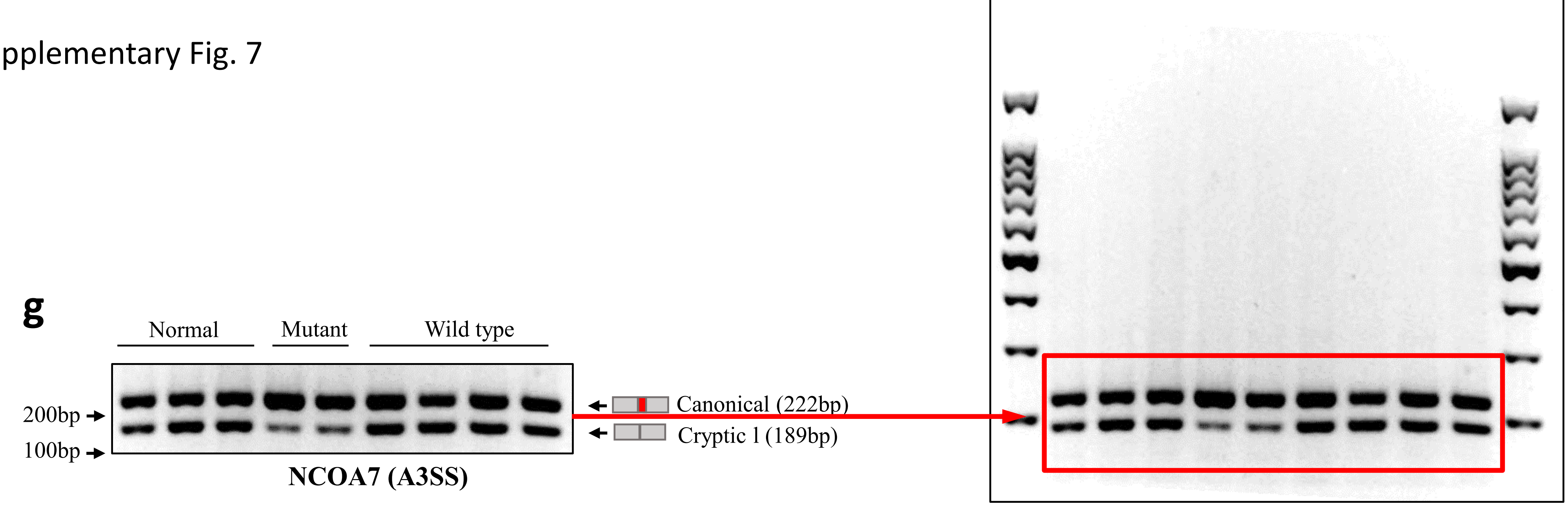

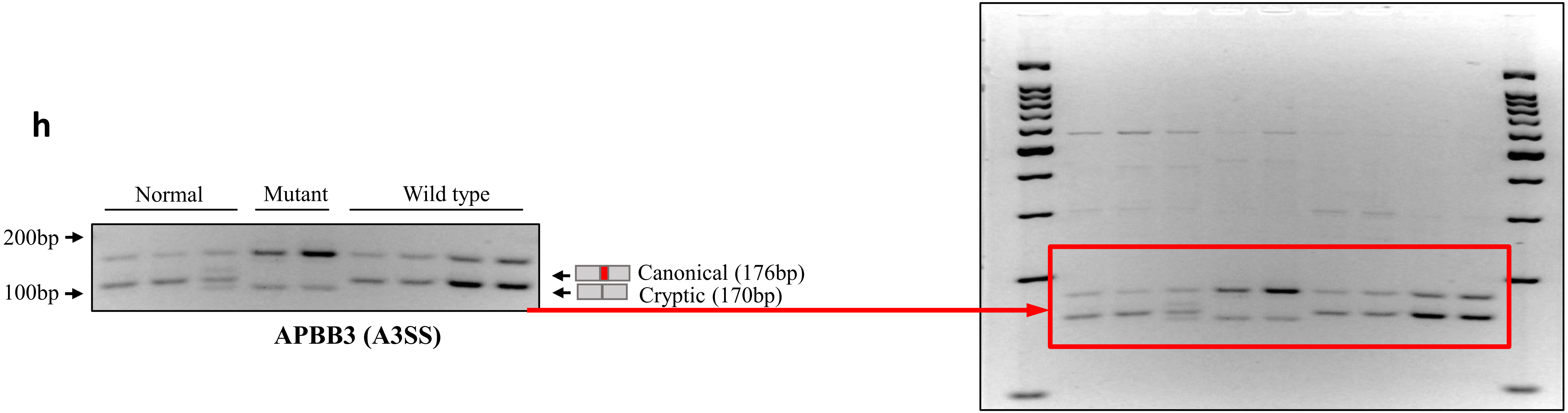

Supplementary Fig. 7

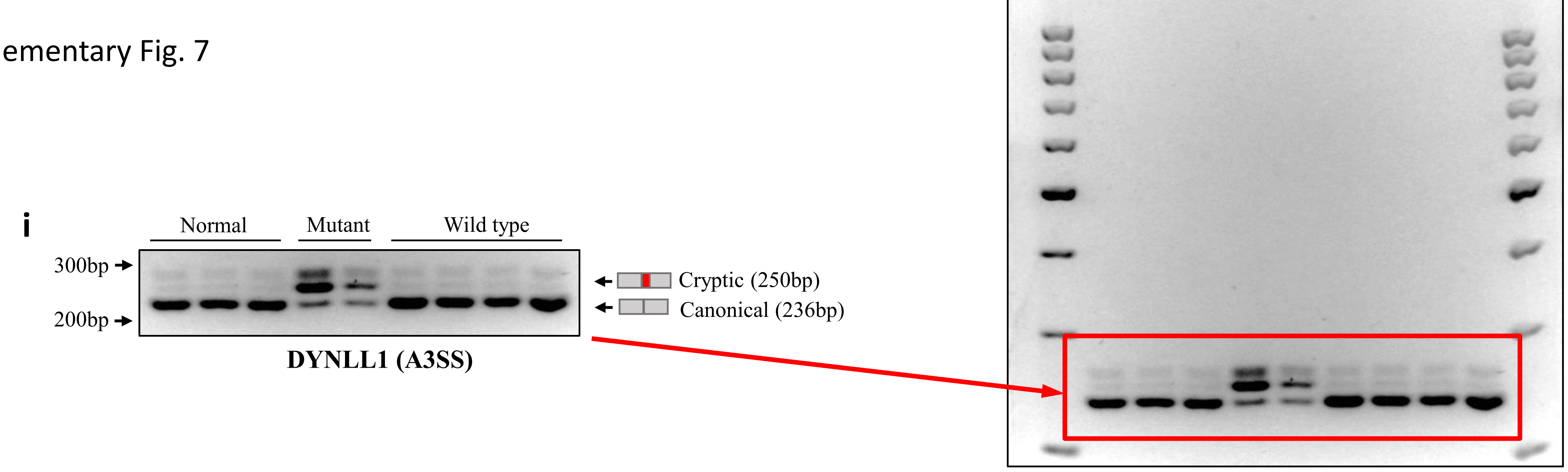

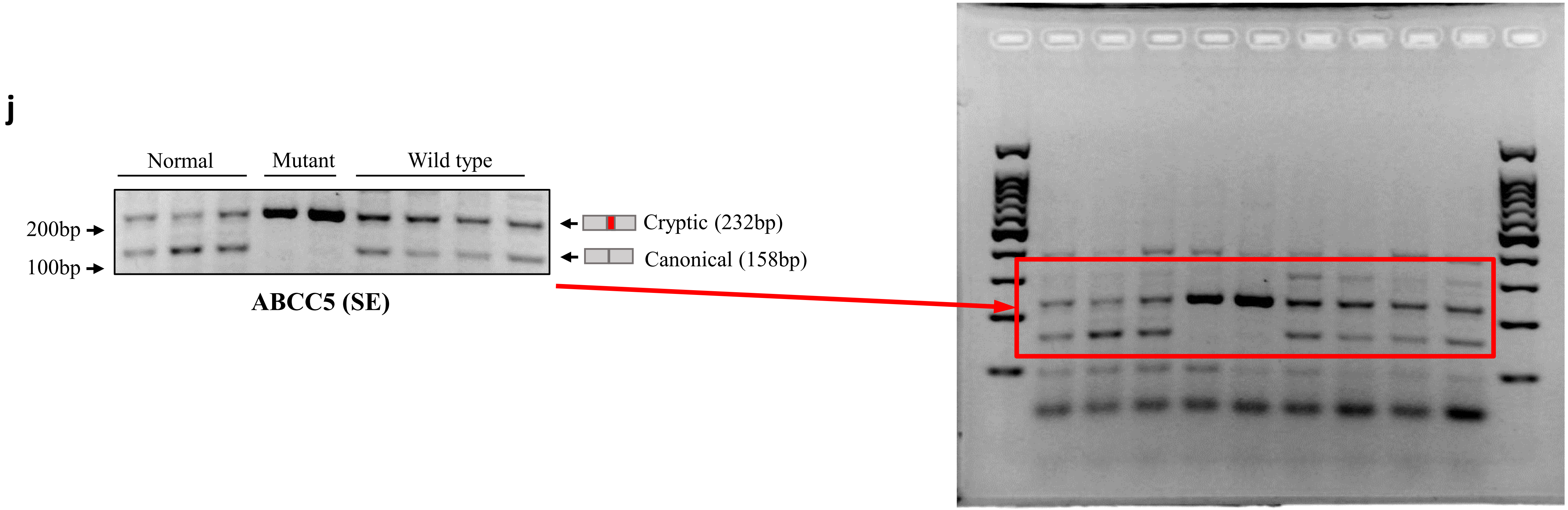

Supplementary Fig. 7

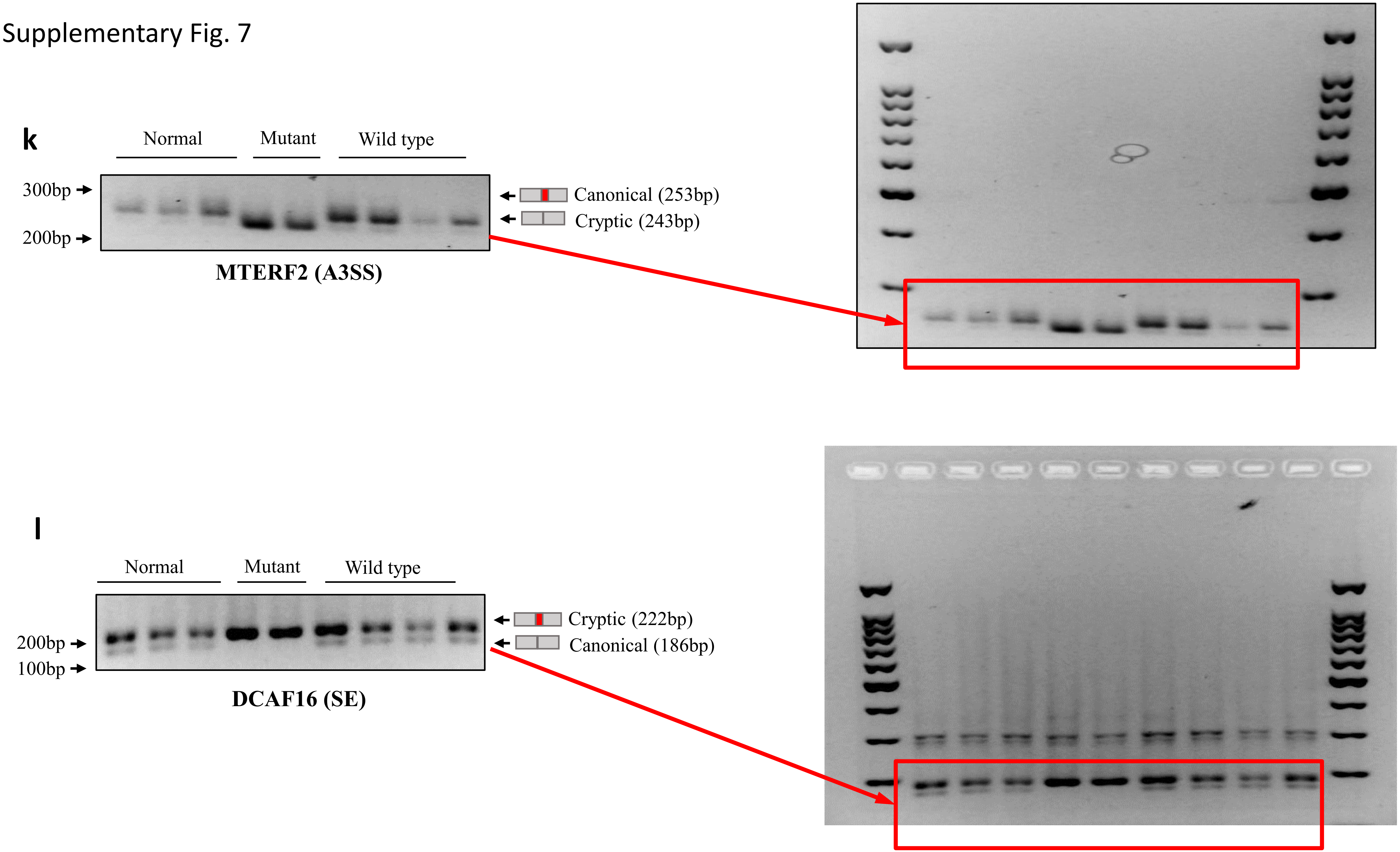

Supplementary Fig. 7

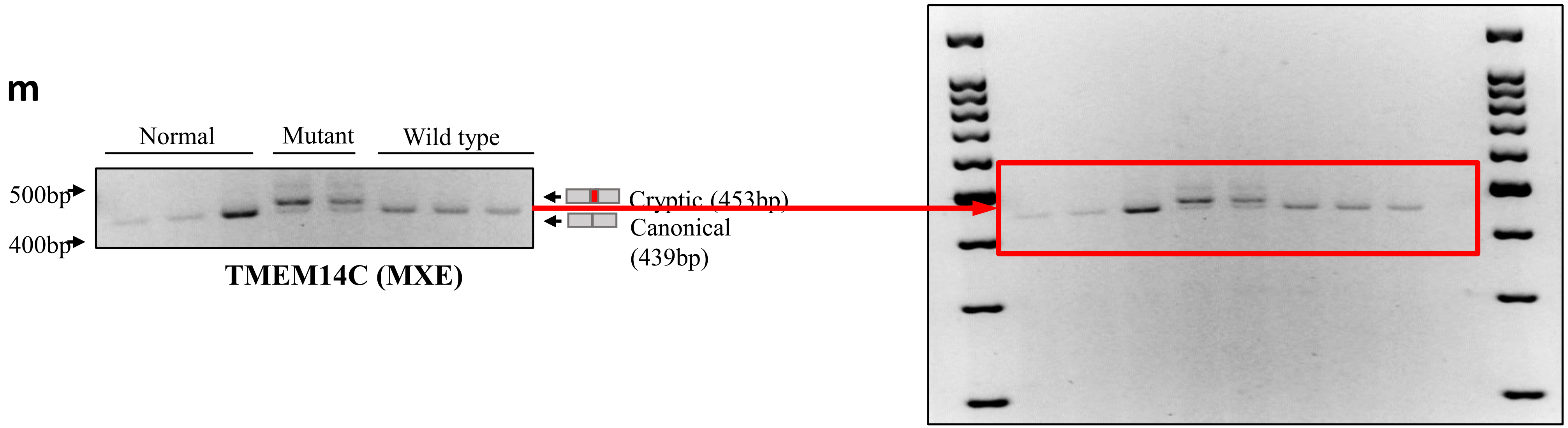

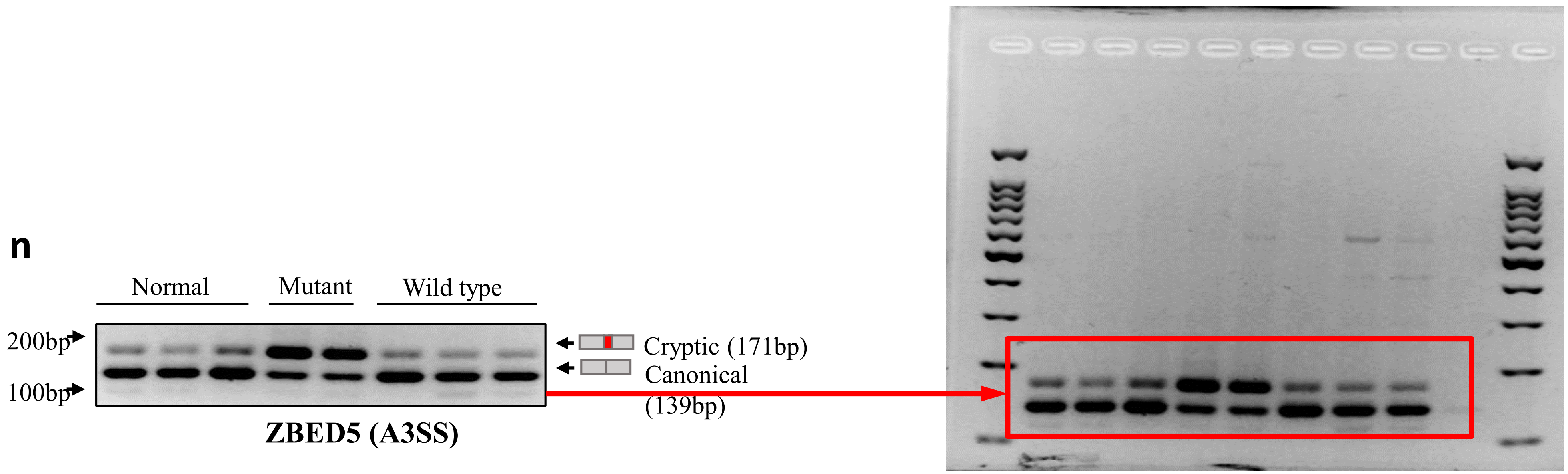

Supplementary Fig. 9a

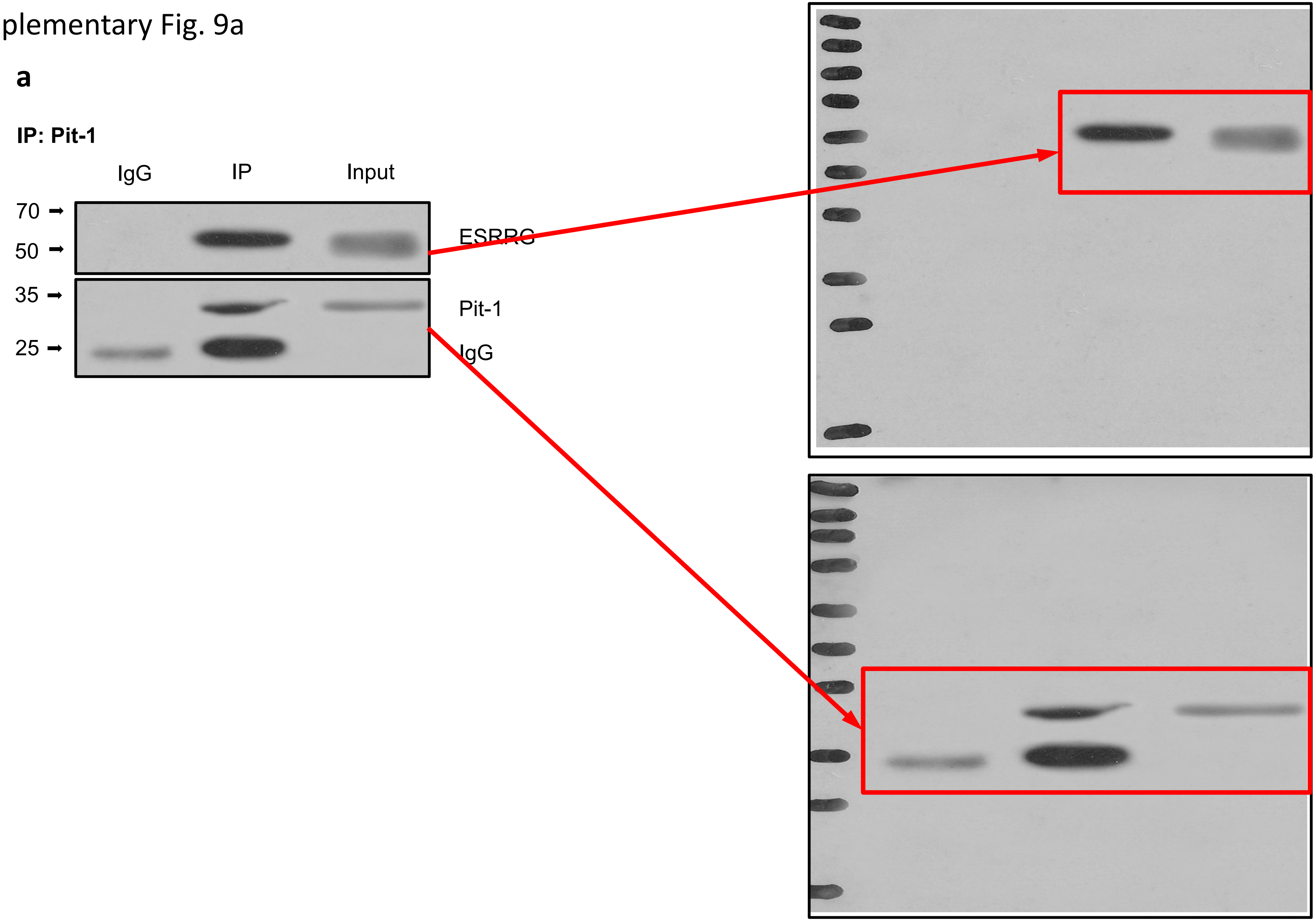

Supplementary Fig. 9b

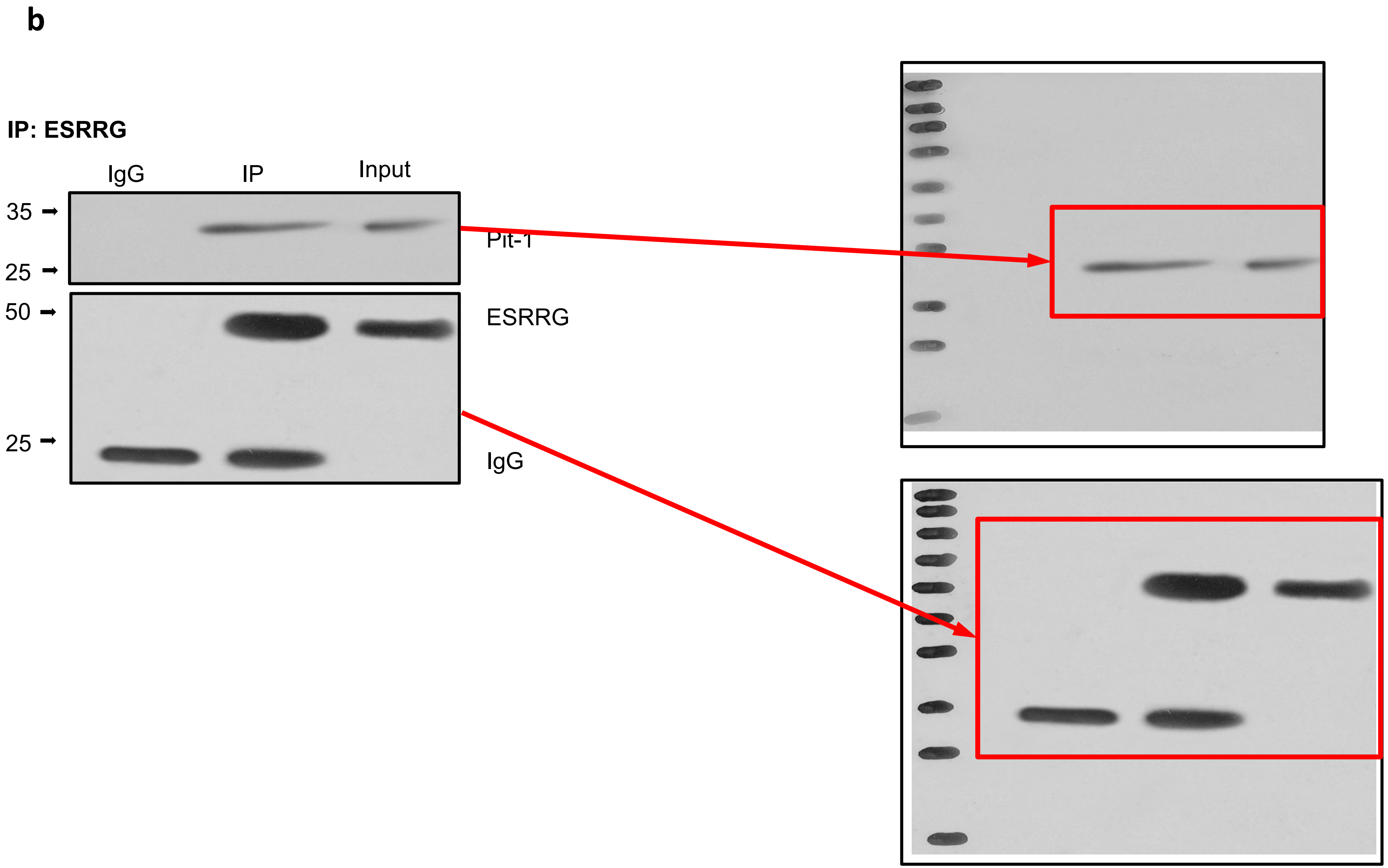

Supplementary Fig. 10

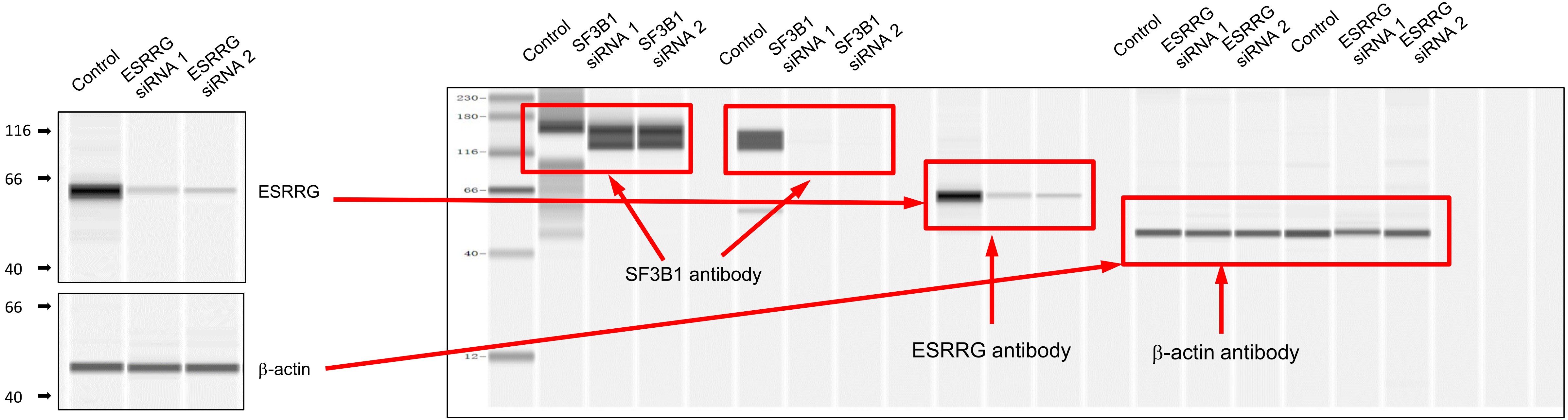

Supplementary Fig. 11

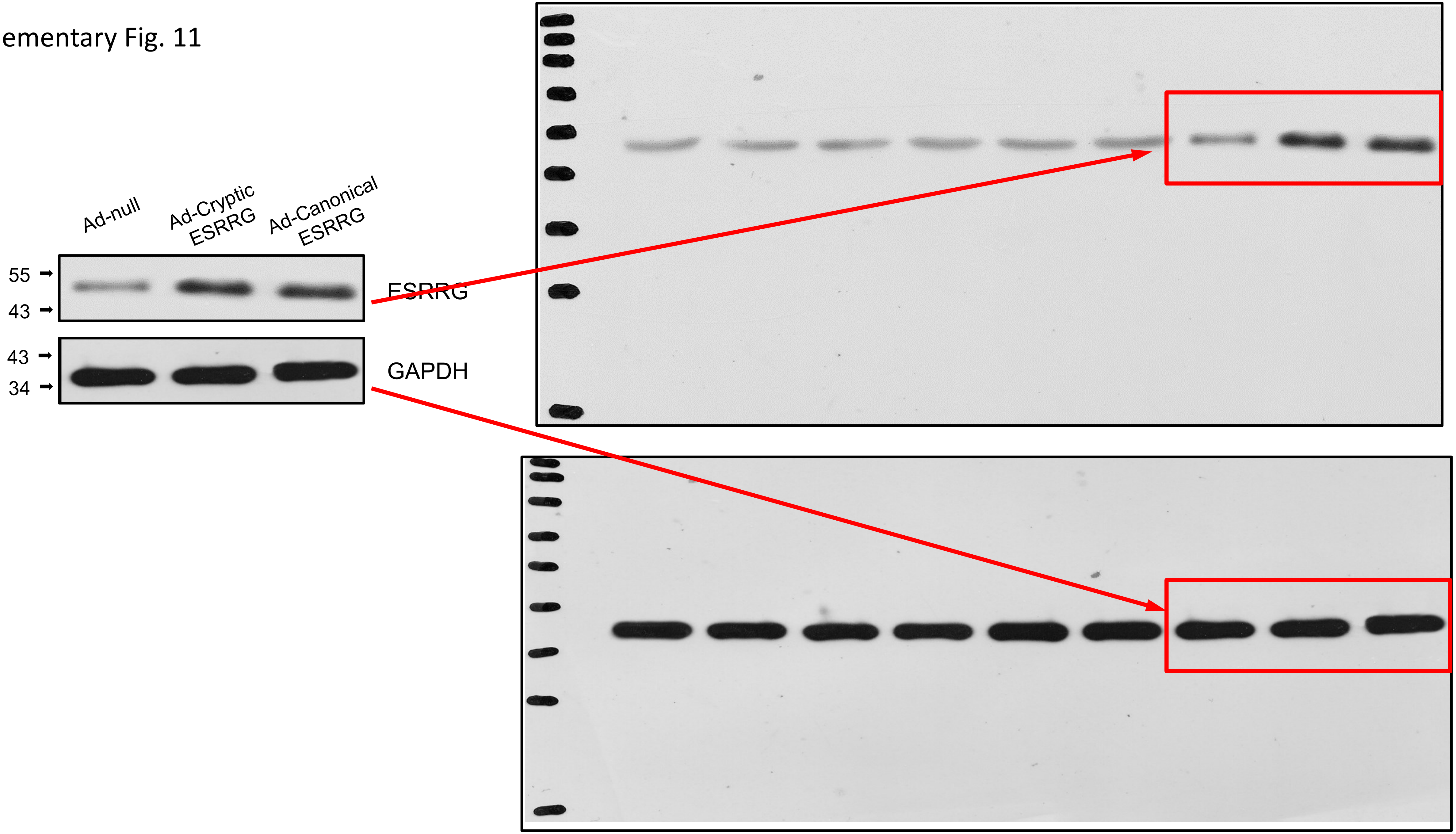

Supplement: Supplementary file 4 — Source Data [file 41467_2020_16052_MOESM4_ESM.zip › 202599_4_related_ms_4528255_q8677k.zip!/202599_4_related_ms_4528255_q8677k.pdf]
